# Comparing Effects in Regular Practice of E-Communication and Web-Based Self-Management Support Among Breast Cancer Patients: Preliminary Results From a Randomized Controlled Trial

**DOI:** 10.2196/jmir.3348

**Published:** 2014-12-18

**Authors:** Elin Børøsund, Milada Cvancarova, Shirley M Moore, Mirjam Ekstedt, Cornelia M Ruland

**Affiliations:** ^1^Centre for Shared Decision Making and Collaborative Care ResearchDivision of MedicineOslo University HospitalOsloNorway; ^2^Department of MedicineUniversity of OsloOsloNorway; ^3^Frances Payne Bolton School of NursingCase Western Reserve UniversityCleveland, OHUnited States; ^4^Royal Institute of TechnologyKTHSchool of Technology and HealthStockholmSweden

**Keywords:** Web-based intervention, electronic mail, Internet, eHealth, cancer, patient-centered care, symptom management, professional-patient relations, randomized controlled trial

## Abstract

**Background:**

While Web-based interventions have been shown to assist a wide range of patients successfully in managing their illness, few studies have examined the relative contribution of different Web-based components to improve outcomes. Further efficacy trials are needed to test the effects of Web support when offered as a part of routine care.

**Objective:**

Our aim was to compare in regular care the effects of (1) an Internet-based patient provider communication service (IPPC), (2) WebChoice, a Web-based illness management system for breast cancer patients (IPPC included), and (3) usual care on symptom distress, anxiety, depression, (primary outcomes), and self-efficacy (secondary outcome). This study reports preliminary findings from 6 months’ follow-up data in a 12-month trial.

**Methods:**

We recruited 167 patients recently diagnosed with breast cancer and undergoing treatment from three Norwegian hospitals. The nurse-administered IPPC allowed patients to send secure e-messages to and receive e-messages from health care personnel at the hospital where they were treated. In addition to the IPPC, WebChoice contains components for symptom monitoring, tailored information and self-management support, a diary, and communication with other patients. A total of 20 care providers (11 nurses, 6 physicians, and 3 social workers) were trained to answer questions from patients. Outcomes were measured with questionnaires at study entry and at study months 2, 4, and 6. Linear mixed models for repeated measures were fitted to compare effects on outcomes over time.

**Results:**

Patients were randomly assigned to the WebChoice group (n=64), the IPPC group (n=45), or the usual care group (n=58). Response rates to questionnaires were 73.7% (123/167) at 2 months, 65.9 (110/167) at 4 months, and 62.3% (104/167) at 6 months. Attrition was similar in all study groups. Among those with access to WebChoice, 64% (41/64) logged on more than once and 39% (25/64) sent e-messages to care providers. In the IPPC group, 40% (18/45) sent e-messages. Linear mixed models analyses revealed that the WebChoice group reported significantly lower symptom distress (mean difference 0.16, 95% CI 0.06-0.25, *P*=.001), anxiety (mean difference 0.79, 95% CI 0.09-1.49, *P*=.03), and depression (mean difference 0.79, 95% CI 0.09-1.49, *P*=.03) compared with the usual care group. The IPPC group reported significant lower depression scores compared with the usual care group (mean difference 0.69, 95% CI 0.05-1.32, *P*=.03), but no differences were observed for symptom distress or anxiety. No significant differences in self-efficacy were found among the study groups.

**Conclusions:**

In spite of practice variations and moderate use of the interventions, our results suggest that offering Web support as part of regular care can be a powerful tool to help patients manage their illness. Our finding that a nurse-administered IPPC alone can significantly reduce depression is particularly promising. However, the multicomponent intervention WebChoice had additional positive effects.

**Trial Registration:**

Clinicaltrials.gov:NCT00971009; http://clinicaltrials.gov/show/NCT00971009 (Archived by WebCite at http://www.webcitation.org/6USKezP0Y).

## Introduction

The number of Web-based support systems to enhance self-management for people living with health conditions has increased rapidly in the last decade, and such interventions have been shown to assist a wide range of patients [1-11]. In cancer care, Web-based support systems are described as helpful for individuals [12]. This includes findings of increased health information competence [13,14], emotional processing [13], fighting spirit [15], social support [14,16], quality of life [16,17], as well as reductions in symptom distress [18,19], and decrease in depression and anxiety scores [17].

However, it can be difficult to distinguish which components of Web-based support systems are most beneficial for patients, and little is known about the relative contribution of different components [13]. In a study of a support system for cancer patients by Baker et al, different features of the system were tested and compared [13]. Results suggested that the benefit of the system was connected to the information (information about cancer, Web links, news, etc) and support services (support from peers and professionals), and that complex services such as coaching and tailoring of content did not produce benefits beyond simple access to the Internet. Another study of the same support system highlighted that benefits depend on *how* a patient uses a system, far more than the total amount of exposure or type of content that is chosen [20]. Overall time spent on the system showed no relation to outcomes. Improvement in patient status was connected to the commitment to use the system over time, independent of how much time they spent on the system. However, high use of communication services (discussion groups and “ask experts or peers”) were associated with decreased negative emotions. Knowledge of the use and effects of single components on patient outcomes will be important to determine component candidates for inclusion in Web-based support systems [13,16,21].

One component often offered as part of multicomponent systems, or as a standalone service, is e-messages. Several studies report benefits from using Internet-based patient-provider communication services (IPPC) for communication between patients and health care providers in terms of assisting patients in managing illness and improving health outcomes [22-24], addressing unmet communication needs [25,26], increasing satisfaction [23,27], and improving quality of care [22,27]. Most of these studies used IPPCs between patients and physicians. In an earlier study of WebChoice, the same Web-based support system for cancer patients used in this study, the nurse-administered IPPC was used by patients to ask questions and raise concerns related to symptom experiences, fear of relapses, and uncertainty in everyday life [18,28]. The IPPC was rated by patients as the most valuable component of WebChoice [29]. High levels of satisfaction with a nurse-administered IPPC were also reported in a study by Cornwall et al [30], but effects on patients’ outcomes of IPPCs alone are rarely described. Thus, we know little about the effect of standalone IPPCs and patient outcomes and how they compare to more comprehensive Web-based support systems where IPPCs are one of several components. In addition, as Web-based support systems require many resources in system development and updating compared to an IPPC, it is interesing to test the effects of these two intervention types compared to usual care.

WebChoice is a Web-based illness management support system based on patient-centered principles and designed to support cancer patients in self-management of their illness, independent of location and time [31]. The purpose of WebChoice is to help cancer patients reduce their symptom distress, improve emotional well-being, and enhance self-efficacy. Results from a previously randomized clinical trial (RCT) that followed 325 breast cancer and prostate cancer patients for 1 year showed that patients with access to WebChoice had significantly reduced symptom distress compared with the usual care control group [18]. Patients in the WebChoice group also had significant within-group improvements in depression during the study period. In addition, the control group experienced significant deterioration in self-efficacy and health-related quality of life during the study. One of the WebChoice features most valued by the study participants, as reported in the previous study, was the opportunity to send e-messages to expert nurses in cancer care, who responded to patients’ questions and concerns within 24 hours [29]. In the RCT described above, WebChoice was offered as a service to patients independent of location and clinical practice. The patients were recruited through advertisements and postal mail. The nurses who answered the e-messages had no treatment responsibilities and did not know the patients. Thus, so far we do not know if similar effects would be achieved if WebChoice or an IPPC were offered as an integrated part of regular care.

As several studies show benefits to Web-based support systems, it is timely to examine the relative contribution of different components of these multicomponent support systems aiming to improve selected outcomes. As cancer patients experience their illness primarily through symptoms, reduction of symptom distress, anxiety, and depression are important indicators of the success of illness management support. Furthermore, there is a need to test the effects of Web-based support as a part of regular care.

The aims of the study were therefore to test and compare the effects of (1) an IPPC, (2) the multicomponent WebChoice intervention (including an IPPC), and (3) usual care (control group) on symptom distress, anxiety, and depression (primary outcomes), as well as self-efficacy (secondary outcome) after 6 months of follow-up. In addition, explorative sub-analyses were performed to detect whether the outcomes were associated with the actual use of the interventions. We hypothesized that the WebChoice group compared with the usual care group would have better outcomes than the IPPC group compared with the usual care group. We also hypothesized that both groups would have better outcomes than the usual care group, on both primary and secondary outcomes.

## Methods

### Subjects and Settings

We conducted an RCT with three groups: two intervention groups (the IPPC service and WebChoice) and a usual care group (clinical trial NCT00971009). Due to slower recruitment than anticipated, we had to stop inclusion after 200 consenting participants, before the calculated sample was obtained. The current paper reports on 167 patients for whom 6-month follow-up data were available at the time this paper was written. These participants were recruited between May 2010 and September 2012.

Inclusion criteria were recent diagnosis of breast cancer treated with surgery, or under treatment with radiation, chemotherapy, hormone therapy, or combinations of those (maximum 12 months after surgery), age over 18 years, able to write/read and speak Norwegian, having access to the Internet at home, and having a public key infrastructure (PKI) solution for secure system access.

Study participants were recruited from three hospitals in Norway—one university hospital and two regional hospitals—at breast diagnostic centers or the ambulatory chemotherapy, radiation, and surgical units. Participants did not receive any incentives for participating in the study.

See Multimedia Appendix 1 for the consent form and Multimedia Appendix 2 for the CONSORT-EHEALTH checklist [32].

### Study Procedures

Eligible patients scheduled for surgery or coming in for checkups after surgery or treatment were identified by the study nurses at the hospitals and provided with information about the study. Upon patients’ arrival at the clinic, the study nurses met the patients, provided brief information about the study, and asked if they were interested in participating. If the patients agreed, the nurse informed them about the study’s purpose and procedures and asked for written informed consent. Consenting patients completed baseline questionnaires before randomization.

After completion of baseline questionnaires, patients were randomized according to a pre-defined automated computerized block randomization, with a block size of 42 stratified by site. Due to the content of the interventions, patients could not be blinded to which arm they were randomized.

Patients randomized to the usual care group were followed up as usual at the hospitals where they were treated. Patients who were randomized into the IPPC or WebChoice groups were informed and instructed in the use of the IPPC or WebChoice. They received a printed user manual with instructions for use, how to log on to the system, and an address and phone number to contact for help if needed. The only in-person information given was instructions on how to access the site and how to connect with the study support service if questions occurred. The study nurses showed them where in the user manual they could go to find information about how to access the site and how to connect with the study support service if needed. In addition, the participants were informed that they could use the IPPC or any component of WebChoice as much or as little as they liked and that using the system was entirely voluntary. The IPPC component was the same for both intervention groups.

After being informed about group assignment, the patients were given access to the interventions the same day. They received an automatic welcome message when the system was ready to use. There was an option to be notified by text message or regular email when new messages appeared in the system. Most participants wanted this notification. All participants were sent questionnaires by postal mail at 2, 4, and 6 months after enrolling.

In total, 20 care providers answered questions from patients: a dedicated group of expert nurses (n=11) and physicians (n=6) in breast cancer care, and social workers (n=3) at the hospital where the patients were treated. They were thoroughly trained in administering the IPPC, technically as well as in codes of conduct for online communication with patients. There was a clear schedule for who was responsible for answering patients’ messages. The nurses were frontline and received all messages first. If necessary, they could forward the message to other care providers. If considered important, information from e-messages could be copied into the medical record and made available for other health care providers. When new questions arrived in the system, the recipient was notified through the hospital’s email system or by text message. The same providers answered e-messages from both the IPPC and WebChoice groups using the same interface. However, they were not entirely blinded to the intervention group assignment because this was sometimes disclosed by patients through the messages. The health care providers had no access to details about how patients used other components of WebChoice. They did not receive any reimbursement or additional dedicated time for answering secure e-messages from the participants.

The study was approved by the Regional Committee for Medical and Health Research Ethics and the Data Security Inspectorate in Norway. Written informed consent was obtained from all participants. All data were submitted to a secure server using an encrypted connection. Patients and health care personnel were authenticated using a public key solution that is currently used by Norwegian banks as a security platform. This means the users’ logon procedure is the same whether they log on to their online bank or to IPPC/WebChoice. Thus, patients did not need to learn a new procedure.

The system experienced a 9-day period of downtime during the first year due to technical problems at the hospital server that hosted the application. No changes were made to the interventions during the trial period apart from fixing minor bugs. The interventions could be used from different Internet browsers and were independent of Internet connection speed.

### Description of the Two Interventions

#### Internet-Based Patient-Provider Communication Intervention

**Figure 1:**
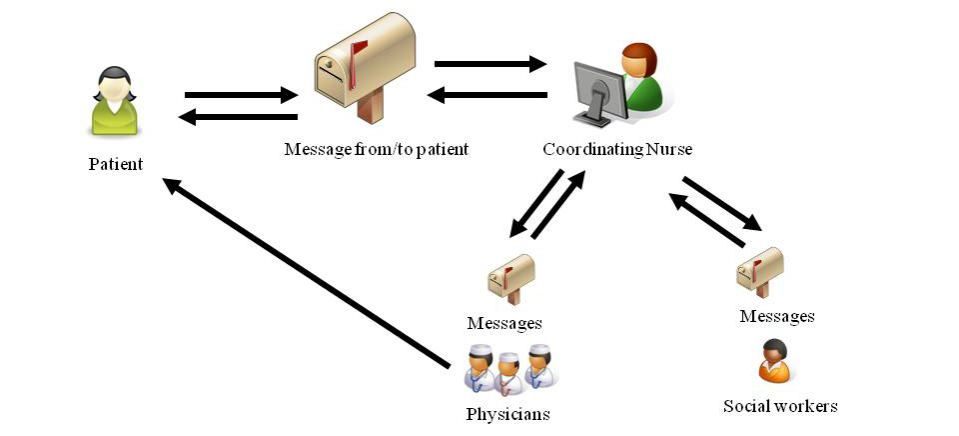
Message flow between patients and health care providers.

The IPPC is a further development of the IPPC component described in a previous study of WebChoice [18,33]. It allows patients to seek help from health care personnel at their treatment hospital. They can ask questions, share experiences with, or get advice from oncology nurses. If needed, the nurse can pass on their question to physicians and social workers (Figure 1). The system has a high security level, where both patients and health care providers log into the system with strong authentication keys. Care providers had access to the patients’ medical records at the hospital. The patient questions were asynchronous and were answered within 2 work days (usually within 1 day).

#### WebChoice Intervention (Internet-Based Patient-Provider Communication Included)

**Figure 2:**
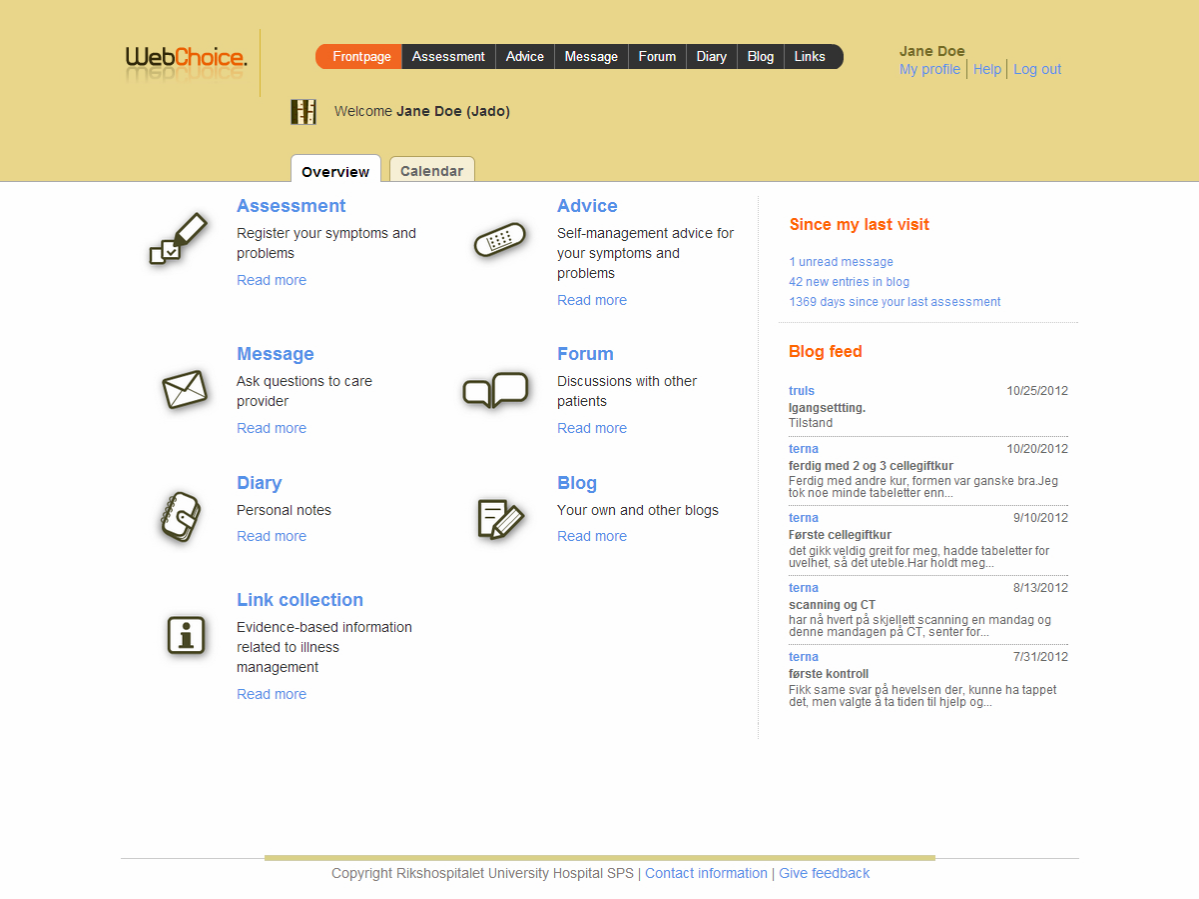
Screenshot of the WebChoice overview page.

WebChoice was developed in close cooperation with users and health care personnel [31]. After testing the system in an RCT [18], it was refined based on responses from users through questionnaires and focus groups. In addition, a blog feature was included. The WebChoice version tested in the current study targeted breast cancer patients and contained the following components in addition to the IPPC service [34] (Figure 2):

1. An assessment component where patients could monitor their symptoms, problems, and priorities for support along physical, functional, and psychosocial dimensions. From a predefined list, patients could choose symptoms and problems they were experiencing, rate the burden of these, and indicate where they needed help. This information could be used to monitor improvement/deterioration of the condition, indicate when to alert health care personnel, prepare for a hospital/physician consultation, improve patient-provider communication, or obtain immediate access to the self-management advice components described below.
2. An advice component provided illness self-management support. The patients’ self-reported symptoms triggered the display of appropriate self-management activities that patients could choose from to relieve symptoms and problems. The component could also be used without finishing an assessment first. Each choice contained an explanation of what the activity was, how to perform it, potential risks, side effects, contraindications, when to contact a physician, levels of evidence, references to the source of the evidence, and links to other reliable websites for related information. The advice component was updated once a year.
3. An information component where patients had access to other reliable Web sources in Norwegian and English, such as information about tests, treatments and potential side effects, lifestyle suggestions, and information about patients’ legal rights. External links were automatically checked every fourth week to ensure they were still active.
4. A communication component for sharing experiences with other patients. Patients could participate in an online forum group discussion that allowed them to exchange messages anonymously with other patients or use a blog. The forum and blog were monitored by nurses at our research center. The nurses did not participate in the forum or blog but answered two postings in the forum not answered by the other participants.
5. An electronic diary where patients could keep personal notes.

### Measures and Data Collection

#### Overview

The primary outcomes were symptom distress, anxiety, and depression. The secondary outcome was self-efficacy. All outcomes were measured at baseline, 2, 4, and 6 months thorough self-assessed questionnaires sent to participants by postal mail.

#### Demographic Characteristics

Patients provided information on age, marital status, level of education, employment status, income, and the use of Internet services.

#### Patient Characteristics

The time of diagnosis and stage of disease were obtained from the medical record. Based on the patients’ tumor (T), node (N), and metastasis (M) classification at the time of diagnosis, the stage of disease was classified into 5 stages (0=ductal carcinoma in situ, to 4=advanced-stage disease) using the TNM Classification of Malignant Tumours of the Union for International Cancer Control guidelines [35].

#### Comorbidity

Patients completed the Self-Administered Comorbidity Questionnaire (SCQ-19), which evaluated the number of, treatments for, and functional impact of health problems. It includes 16 common comorbidities and three optional conditions [36]. The total SCQ-19 score can range from 0-57 when the three optional items are used. It is a clinical scale, with established validity and reliability [36], for the assessment of comorbidities in patients with chronic medical conditions. A higher total score indicates a more severe comorbidity profile.

#### Symptom Distress

Symptom distress was measured by using the 32-item Memorial Symptom Assessment Scale (MSAS) [37], which lists physical and psychological symptoms that occur due to cancer or its treatment. For each symptom, patients were asked to indicate whether they had had the symptom during the previous week. If they had experienced the symptom, they were asked to rate its frequency, severity, and distress. Symptom frequency and severity was rated using a 4-point Likert scale. Symptom distress was rated using a 5-point Likert scale. The reliability and validity of the MSAS are well established [37], and MSAS has previously been used in breast cancer populations [38]. Higher scores indicate greater symptom distress. Cronbach alpha coefficient for our sample at baseline was .85.

#### Anxiety and Depression

Anxiety and depression were measured with the Hospital Anxiety and Depression Scale (HADS) [39], a 14-item, self-report measure of psychological distress. The HADS is divided into 2 subscales: anxiety (HADS-A) (7 items) and depression (HADS-D) (7 items). Respondents are asked to indicate which of 4 response options (rated from 0-3; score range, 0-42) comes closest to describing how they have been feeling in the previous week for each item. Scores from 0-7 on the subscales are regarded as being in the normal range; a score of 11 or higher indicates a probable presence of a mood disorder, and a score of 8-10 is suggestive of the presence of the state [40]. The scale is found to perform well in assessing the symptom severity of anxiety disorders and depression in hospital settings, in primary health care and in the general population [41], and has demonstrated acceptable reliability in cancer populations [42]. Cronbach alpha coefficient is reported to vary from .68-.93 for HADS-A, and for HADS-D from .67-.90 [41]. In our sample, Cronbach alpha at baseline was .83 for HADS-A and .76 for HADS-D.

#### Self-Efficacy

Self-efficacy was measured with the Cancer Behavioral Inventory (CBI) version 2.0 [43], a 33-item instrument that measures coping self-efficacy with cancer-related stress on 7 dimensions: (1) maintenance of activity and independence, (2) seeking and understanding medical information, (3) stress management, (4) coping with treatment-related side effects, (5) accepting cancer and maintaining a positive attitude, (6) affective regulation, and (7) seeking support. Responses on 9-point Likert scales ranged from 1 (not at all confident) to 9 (totally confident). Higher scores indicated greater self-efficacy. CBI was used in a previous study testing WebChoice among breast and prostate cancer patients [18] and, according to Merluzzi et al, has good internal consistency with a Cronbach alpha coefficient reported of .94 [43]. Cronbach alpha coefficient for our sample at baseline was .96.

### System Use

Data on system use were extracted from the user logs on the server. Information was collected on how many times the users had logged on and which components of WebChoice were accessed or used actively.

### Analysis

#### Overview

Data on baseline characteristics are presented as medians and ranges for continuous variables and as proportions with percentages for categorical data. Differences between users and non-users were analyzed using the chi-square test for pairs of categorical variables. The Mann-Whitney-Wilcoxon test was used for continuous data with skewed distributions.

#### Effectiveness

For analysis of between-group differences in symptom distress, anxiety, and depression (primary outcomes) and self-efficacy (secondary outcome), linear mixed models (LMM) for repeated measures were fitted. A diagonal covariance structure was used to model dependencies among measurements on the same individual at different time points. Models for each outcome consisted of 3 effects: measurement occasion (time), interventions (WebChoice, IPPC, usual care), and the interaction of time and intervention. All measured time points of the outcome variables are considered and the LMM approach therefore adjusts for baseline differences. To test whether potential confounders impacted the results, LMM adjusted for variables such as site, age, marital status, education, time since diagnosis, stage of disease, and comorbidity were fitted. Compared to the unadjusted models, these adjusted models revealed even larger differences in favor of the intervention groups compared to the usual care group. Taking the limited sample size into account and aiming to avoid over fitting, only the results from the unadjusted models are presented. As no statistically significant differences were observed between the study groups on demographic and disease-related factors at baseline, these models were not further adjusted for the possible confounders. The authors are aware that this might underestimate the true differences between the groups. Analyses of primary and secondary outcomes were conducted on an intention-to-treat basis, including all participants in each group, independent of whether they were users or non-users of the interventions. The model parameters are estimated using the classical maximum likelihood approach. No imputation of missing data was necessary or performed, as the LMM uses all data available to estimate the covariance matrix and model the dependencies. The results are presented as *P* values for the overall effect of the variables when baseline score and all time points are included. Moreover, overall mean differences are presented, that is, the difference between groups adjusted for baseline scores and taking all time points into consideration. Report of overall mean differences was chosen as we were interested in differences between the groups over the entire 6-month period.

#### Explorative Sub-Analyses

In addition, explorative sub-analyses were performed to detect whether the outcomes were associated with the actual use of the interventions. LMM for repeated measures were fitted. Models for each outcome were fitted with three factors: measurement occasion (time), interventions (user/non-user of WebChoice and IPPC), and the interaction of time and intervention. Age was added as a covariate because age is known to be associated with use of Web-based tools [44,45].

Analyses were carried out using SPSS version 18.0. *P*-values <.05 were considered statistically significant, and all tests were two-sided.

## Results

### Participation Rates

**Figure 3:**
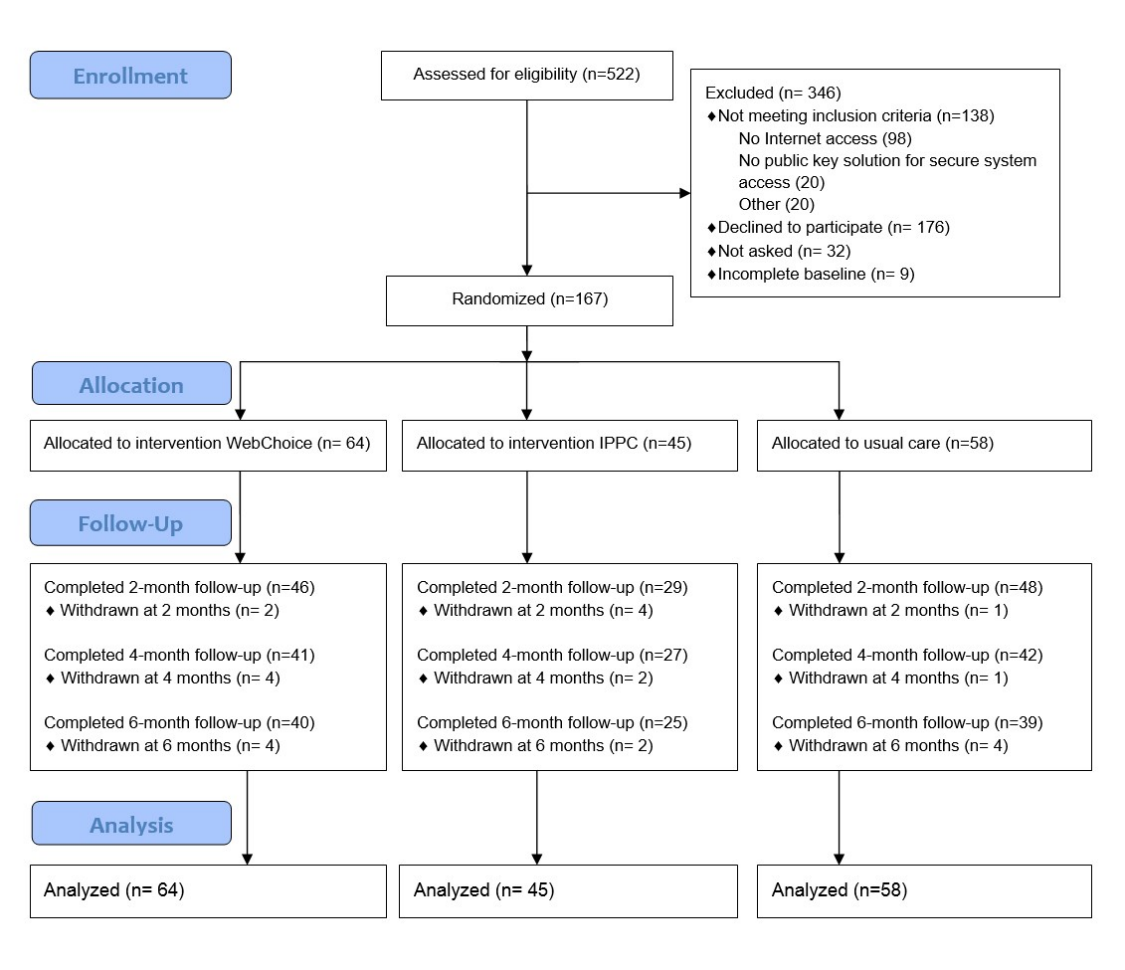
Flow of participants throughout the trial.

The trial flow chart (Figure 3) shows the recruitment and retention at baseline and at 2-, 4- and 6-month follow-up. From May 2010 to September 2012, 522 breast cancer patients were assessed for eligibility. Of these, 138 did not meet the inclusion criteria, mostly due to lack of Internet access, and 176 declined to participate. Non-participants were slightly older; the median age among those who did not meet the inclusion criteria was 67 years and among those who declined to participate, 59 years. Frequent reasons given for declining were lack of experience with computers/ Internet or that they had too much on their mind related to their illness. The 176 patients who agreed to participate in the study were randomized after filling in baseline questionnaires. Nine patients were excluded due to incomplete baseline data, leaving a sample of 167. During 6 months of follow-up, we had a 14% (23/167) attrition rate. Information on reasons for withdrawal is not available. There was no association between baseline characteristics and those who left the study. At the 6-month measurement, 62% (104/167) answered the questionnaires: WebChoice 62% (40/64), IPPC 57% (25/45), and usual care group 67% (39/58).

### Baseline Characteristics

There were no statistically significant differences in demographics, disease-related factors, or outcome measures between participants in the three groups at baseline (Table 1).

**Table 1:** Baseline demographic and illness characteristics (n=167).

Characteristics		WebChoice (n=64)	IPPC (n=45)	Usual care (n=58)	*P* value
Age in years, median (range)		51 (37-79)	50 (31-66)	53 (36-69)	.21
**Marital status, n (%)**					.63
	Married/cohabitating	52 (82)	34 (76)	43 (77)	
	Single/divorced	11 (18)	11 (24)	13 (23)	
**Education, n (%)**					.29
	Elementary/high school	23 (37)	22 (49)	30 (54)	
	University/college ≤4 years	24 (39)	14 (31)	12 (21)	
	University/college >4 years	15 (24)	9 (20)	14 (25)	
**Household annual income, (NOK** ^a^ **), n (%)**					.46
	<400,000	8 (13)	12 (27)	10 (18)	
	400,000 to 600,000	14 (23)	9 (20)	18 (32)	
	600,000 to 800,000	19 (31)	11 (24)	13 (23)	
	>800,000	21 (34)	13 (29)	15 (27)	
**Employment status, n (%)**					.54
	Full-time/part-time work	21 (33)	18 (41)	14 (26)	
	Sick leave/disability benefits	34 (54)	22 (50)	32 (58)	
	Retired/other	8 (13 )	4 (9)	9 (16)	
**Stage of disease, n (%)**					.53
	0	2 (3)	1 (2)	5 (9)	
	1	24 (38)	21 (47)	26 (45)	
	2	32 (50)	20 (47)	24 (41)	
	3	6 (9)	2 (4)	3 (5)	
**Psychosocial factors, median (range)**					
	Symptom distress	.47 (.03-1.49)	.52 (.03-1.76)	.59 (.05-1.86)	.36
	Anxiety	4.0 (0-16)	5.0 (0-17)	4.0 (0-15)	.38
	Depression	1.5 (0-11)	2.0 (0-10)	2.0 (0-14)	.37
	Self-efficacy	247 (110-297)	214 (101-297)	238 (102-297)	.11
Months since diagnosis, median (range)		1 (0-10)	0 (0-9)	1 (0-10)	.16
Comorbidity, median (range)		2 (0-10)	2 (0-16)	2.0 (0-13)	.51
**Sending/receiving email, n (%)**					
	> 1x/week	57 (91)	37 (82)	53 (95)	.09
	< 1x/week	2 (3)	6 (13)	3 (5)	
	< 1x/month	4 (6)	2 (4)	0 (0)	
**Reading information on Internet, n (%)**					.28
	> 1x/week	54 (87)	36 (80)	43 (78)	
	< 1x/week	3 (5)	2 (4)	7 (13)	
	< 1x/month	5 (8)	7 (16)	5 (9)	
**Participation in social media/groups, n (%)**					.67
	> 1x/week	28 (45)	18 (40)	20 (36)	
	< 1x/week	7 (11)	9 (20)	8 (15)	
	Never	27 (44)	18 (40)	27 (49)	

^a^NOK=Norwegian kroner: 400,000≈$US 67,000; 600,000≈$US 100,000.

### Effectiveness

**Figure 4:**
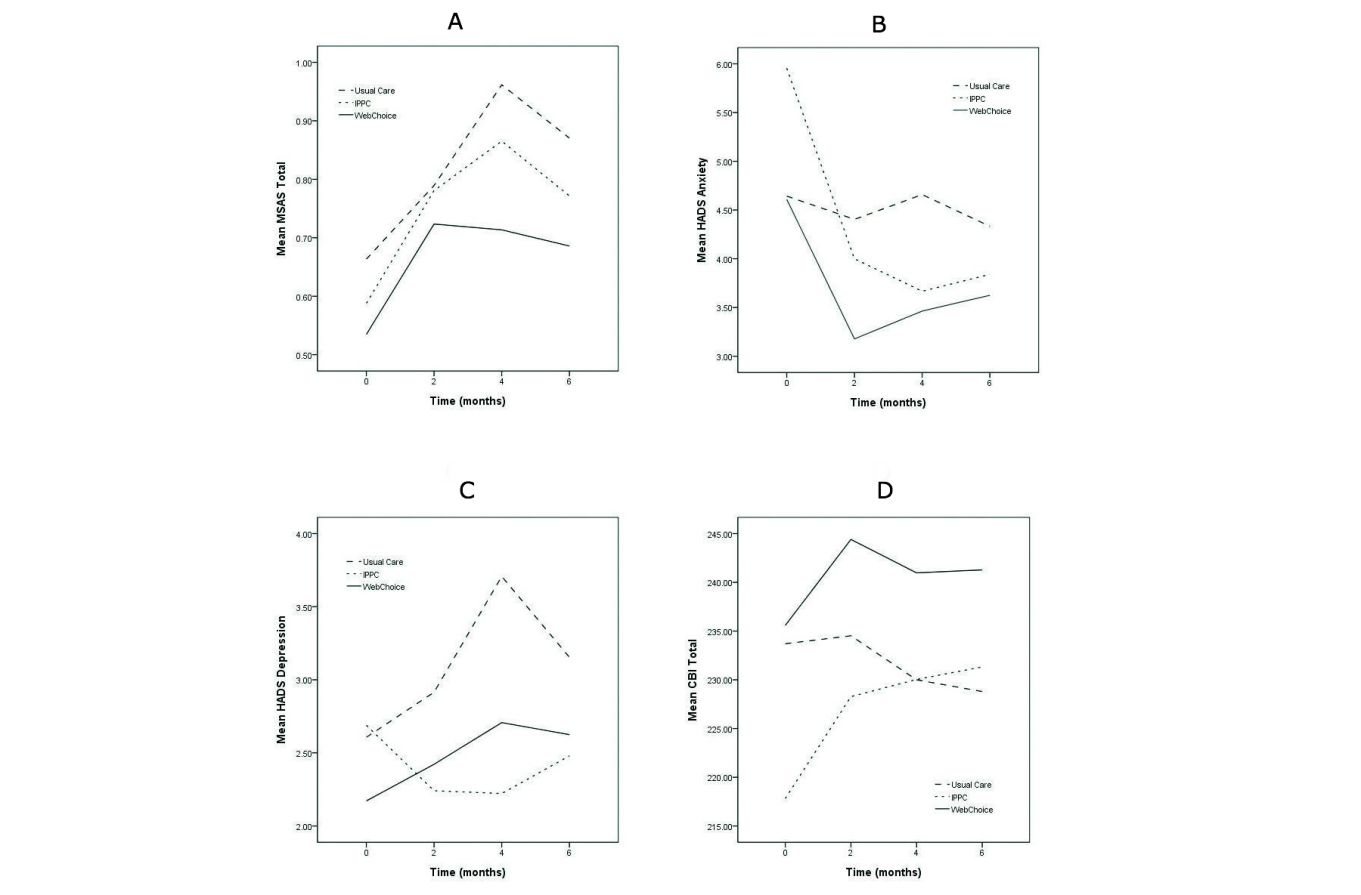
Estimated marginal means of (A) symptom distress (MSAS Total), (B) anxiety (HADS Anxiety), (C) depression (HADS Depression), and (D) self-efficacy (CBI Total) for the usual care group (n=58), the IPPC group (n=45), and the WebChoice group (n=64).

Figure 4 and Table 2 provide the patients’ self-reported scores for the primary outcomes of symptom distress, anxiety, and depression. When measurements at all four time points were included in the model, the WebChoice group reported significantly lower symptom distress over time (mean difference -0.16, 95% CI -0.25 to -0.06, *P*=.001), anxiety (mean difference -0.79, 95% CI -1.49 to -0.09, *P*=.03), and depression (mean difference -0.79, 95% CI 1.18 to -0.05, *P*=.03) compared with the usual care group. Over time, the IPPC group had significantly lower depression scores compared with the usual care group (mean difference -0.69, 95% CI -1.32 to -0.05, *P*=.03), but no differences were observed for symptom distress or anxiety. Time by condition interactions was examined, but no significant results detected. There were no statistically significant differences over time between the two intervention groups on symptom distress, anxiety, and depression (data not shown).

The WebChoice group tended to score higher than the usual care group on self-efficacy (secondary outcome) over time (mean difference 8.81, 95% CI -0.92 to 18.53, *P*=.08) (Table 2 and Figure 4). No statistically significant differences were found over time between the IPPC group and the usual care group on self-efficacy.

**Table 2:** Effect of IPPC (n=45) and WebChoice (n=64) compared with the usual care group (n=58) on outcome variables: summary of basic mixed models.

	WebChoice			IPPC		
	Mean diff^a^	95% CI	*P* value	Mean diff^a^	95% CI	*P* value
MSAS total	-0.16	-0.25 to -0.06	.001	-0.07	-0.18 to 0.04	.21
HADS-A	-0.79	-1.49 to -0.09	.03	-0.14	-0.93 to 0.64	.72
HADS-D	-0.61	-1.18 to -0.05	.03	-0.69	-1.32 to -0.05	.03
CBI	8.81	-0.92 to 18.53	.08	-4.89	-15.90 to 6.12	.38

^a^Based on estimated marginal means. Analyses adjusted for baseline scores.

### System Use

In accordance with the CONSORT-EHEALTH checklist [32], we analyzed the use of IPPC and WebChoice among those who had access to the applications. Among those randomized to the IPPC, 40% (18/45) sent at least an e-message and were defined as users (Table 3). In the WebChoice group, 77% (49/64) logged on at least once during the 6 months, and 39% (25/64) sent e-messages. We defined the two-thirds (41/64) who accessed WebChoice more than once as users. We required patients to log in at least twice before we defined them as users because patients who logged in only once may have read only the welcome message and never actually used the system. The users logged on a median 7 times (range 2-41).

Table 3 shows the use of the IPPC and the usage of different components in WebChoice among the users. Of the WebChoice users, 61% (25/41) sent e-messages, 20% (8/41) posted in the discussion forum, and 37% (15/41) posted their own blogs. However, patients visited the IPPC, forum, and blog more often to read information without submitting their own postings. Reading of other blogs was the component most highly accessed (total 684; range 0-58, median 14). The advice components were also highly accessed (total 317; range 0-62; median 5).

The IPPC messages were mainly answered by nurses within their regular working hours. Of the 153 messages sent by the respondents, 22% (33/153) were passed on to and answered by physicians. Only one message was passed on to social workers. Patient questions posed through the IPPC were always answered via secure e-messages. In a few instances, the nurses informed the patient that they could contact her by phone to discuss issues voiced in the message. Time spent on answering messages was not measured. However, there were no indications that the nurses or physician felt the e-message answering task had been too time consuming for them during the study.

There were no differences between users and non-users in either intervention group on any demographic or disease-related variables.

**Table 3:** Usage of components in WebChoice (n=41) and IPPC (n=18) over 6 months of access.

	Users WebChoice (n=41)					Users IPPC (n=18)				
Components	Times accessed			Users who accessed at least once		Times accessed			Users who accessed at least once	
	Median	IQR^a^	Range	n	%	Median	IQR^a^	Range	n	%
Total visits	7	11	2-41	41	100	9.5	10	1-17	18	100
E-messages sent	1	3	0-9	25	61	3.5	4	1-7	18	100
Total e-messages visits	4	7	0-25	39	95	12	12	2-24	18	100
Assessments	1	2	0-7	30	77					
Assessment visits	5	6	0-25	40	98					
Advice views	5	10	0-62	29	71					
Information section visits	2	3	0-28	34	83					
Posts in forum	0	0	0-3	8	20					
Forum visits	4	4	0-25	38	83					
Posts in blog	0	2	0-9	15	37					
View of others’ blogs	14	19	0-58	37	90					
Diary notes	0	3	0-14	19	46					
Diary visits	3	6	0-26	35	85					

^a^IQR=interquartile range.

### Exploratory Analysis: Effect of System Use on Outcomes

No differences were detected between users and non-users of WebChoice or IPPC on symptom distress, depression, or self-efficacy (data not shown). The users of the IPPC had significantly lower scores on anxiety compared with the non-users (mean difference -1.28, 95% CI -2.54 to -0.01, *P*=.047). No such differences between users and non-users were observed in the WebChoice group (data not shown).

## Discussion

### Principal Results

The current effectiveness study demonstrates that access to the multicomponent Web-based support system WebChoice for 6 months, among women with breast cancer within the first year after diagnosis, reduced symptom distress and levels of anxiety and depression scores. A tendency towards increased self-efficacy could also be detected for the WebChoice group. This is promising given three diverse practice settings in regular care. Also noteworthy is the finding that access to an IPPC alone reduced depression scores. These results support the hypothesis of the WebChoice group having better outcomes than the IPPC group in symptom distress and anxiety, compared to the usual care group. The IPPC group had a similar effect on reduction of depression as WebChoice, however, and the WebChoice group did not have better self-efficacy than the IPPC group compared to usual care.

Our findings are in line with previous research showing that Web-based support systems in cancer populations can decrease depression and anxiety scores [17] and reduce symptom distress [18,19]. The reported effects in our study were detected despite a smaller sample size than initially calculated. However, in this study participants were more homogenous compared with the previously published study [18] that included both women with breast cancer and men with prostate cancer. In addition, the previous study included patients with recurrence of disease and metastasis, and patients were included independent of time since diagnosis (mean time since diagnosis 2.2 years), whereas patients in our study were included within the first year after diagnosis (median time since diagnosis 1 month).

One possible explanation for our significant results might be that both WebChoice and IPPC might meet unmet needs reported by cancer patients, such as needs within the communication, information, psychosocial, psychological, and supportive care domains, generally highest during the treatment phase [46]. WebChoice allows patients to monitor their psychological, psychosocial, and physical symptoms and also get individually tailored information and support on how to manage their symptoms through the advice component. The information component can offer educational information through access to other reliable Web sources. Through WebChoice, patients are also able to read the information repeatedly when it suits them. Other studies suggests that Web-based information sources are used for different purposes [47] and that it can be easier to address sensitive information through email service than in personal encounters with health care professionals [25]. Offering self-management interventions in this early phase might be especially helpful. The need for early interventions was also supported by a study of an IPPC service similar to the one in the current study, where the need for such a service was described as being most prominent during the first phase after discharge from hospital [25].

Our results indicating that the IPPC reduced depression scores are especially promising, as depression is one of the most debilitating symptoms people can have and is highly prevalent among cancer patients [48,49]. This result was detected despite respondents being recruited from different settings with variations in organization of care, which holds promise for IPPCs as effective interventions for reduction of depression scores among breast cancer patients across settings.

The IPPC’s ability to reduce depression scores might relate to indications that patients with higher scores of depression, in addition to higher symptom distress and low social support, are high users of the IPPC service in WebChoice [47]. As such, the IPPC is an intervention that is used by those with high illness burden, a group with high needs, and potential for improvement. The median baseline scores of both anxiety and depression were below the defined cut-off scores of 8, which is predictive for presence of anxiety and depression [40,41]. However, reduction of scores indicates that people are feeling better. Our limited sample size did not allow analyses on how the intervention affected the individuals who had scores of 8 or more.

Whether the differences in effects of the IPPC and WebChoice are related to the additional features contained in WebChoice is not clear. The IPPC feature might be used differently in the two groups, and as such we were not able to fully disentangle the effect of the IPPC feature. The patients would have had to be allocated to separate conditions in order to test different features. However, as in the IPPC group where 40% (18/45) sent e-messages, 39 % (25/64) in the WebChoice group sent e-messages, suggesting that interest in using this component is the same, independent of other features available.

### Use and Relation to Effects

Use was not connected to the observed effects on symptom distress and depression in the current study. Similar results were observed in the previous study of WebChoice [18]. The findings that IPPC users had significantly lower scores on anxiety (*P*=.047) than the non-users must be interpreted with caution. The groups compared were small (18 users and 27 non-users), and thus the finding might be spurious. WebChoice is an illness management support system designed to support cancer patients in self-management of their illness. The system offers different components, so participants can use what they prefer without any push to use the system. Our findings of positive effects in the study despite user frequencies of just 64% (WebChoice) and 40% (IPPC) might relate to the psychological effects of the sheer possibility of using the system when needed. Interviews with non-users of a similar IPPC revealed that even if they did not use the system, they liked having the possibility [50]. The assurance that someone is available and can answer the questions important to a patient may contribute to the effects observed on depression in the current study. The opportunity to get the information needed for self-management of symptoms and problems, independent of time and location, might also contribute to the other findings of reduced symptom distress and anxiety.

### Limitations

Several limitations need to be addressed. The first limitation concerns the small sample size. A larger sample would have increased the validity of the study but would also have prolonged the time needed for recruitment. In the recruitment process, most of the potential participants were approached. However, only a third of those approached were included. Lack of access to Internet, the most frequent reason for not meeting the inclusion criteria, was reported by 19% (98/522) of those approached. Among those who were eligible, a frequent reason given for declining participation was that patients judged their computer and Internet skills as poor. One way to increase the participation rates might be to offer a demonstration of the interventions at the time of inclusion.

A smaller sample size than initially calculated and the attrition rate during the study reduced statistical power for our analyses. Because we had to stop inclusion of participants before the a priori calculated sample was obtained, block randomization led to different sample sizes in the three groups, with the least participants in the IPPC group. The project was also subject to high attrition during the study, which is not uncommon in studies of eHealth interventions [51]. In addition, the IPPC group had the lowest number of completers of questionnaires at 6 months. Our analysis should thus be interpreted with caution, and additional research is needed to confirm our results.

Another limitation relates to low use of the interventions. The analyses of the intervention groups compared to the usual care group therefore compare the effects of a little used intervention. However, as the effects were detected through intention to treat analysis, the effect might be connected to the option and possibility of using the system, not necessarily to actual use. Our post hoc analyses of usage and its relation to outcomes were based on a smaller sample, comparing users and non-users, and must be viewed as an exploratory analysis only.

Interventions such as WebChoice, which offer components where people interact with each other (forum and blog), need a critical mass in order to be fully utilized. A study period of 6 months, an inclusion period of nearly 2.5 years, and the three-armed study design resulted in few participants receiving the WebChoice intervention simultaneously. This could be an explanation for low use of the discussion forum in this study.

Patients included in the study were younger (median age 52) than those who were excluded (median age 67) or who declined to participate (median age 59). In addition, the participants had higher education levels than the average level of education in Norway, suggesting that they were not representative of all age and educational groups among breast cancer patients. This, together with the small sample size, rate of declining participation, and the attrition from the study likely lowers the generalizability of the findings. On the other hand, the patients were recruited from three different hospitals across the country, which increased generalizability of findings across practice settings. Finally, nearly 50% of the participants were included right after the time of diagnosis. However, the other half was included at 1-10 months after diagnosis. As such, they were in different phases in their treatments and experienced different side effects at the time of questionnaire completion. This might have influenced the symptoms reported on the MSAS and HADS. On the other hand, this variability might strengthen our results.

### Implications

This study illustrates the feasibility of offering parts of Web-based support systems in regular care, as the IPPC components were answered by nurses/physicians at the hospital where the patients were treated, providing the patients with easy access to the expertise, without a face-to-face appointment. The IPPC service, with its ability to reduce depression scores, will be an important component to integrate in Web-based support systems and can also be offered as a standalone system.

The integration of Web-based support into clinical practice will require some changes, and changing routines in care is challenging [52-54]. There are reports of skepticism among care providers about use of IPPCs in routine care [55,56]. Some health care providers have expressed concerns that the use of e-messages might disrupt existing workflows and increase workloads [55]. Patients, however, expect to be able to communicate with their health care providers through e-messages [27,55,57], and integration of the IPPC (as a standalone service or as part of multicomponent support systems) does not require a huge change in health care routines. In this study, only a few nurses and physicians were trained to answer the IPPC. The number of e-messages in the study was moderate and most were answered by nurses, during their regular working hours. As such, the IPPC did not interfere with the workflow of the entire staff, and the number of e-messages was reported as manageable. The nurses answering the IPPC performed their new task during regular working hours, without any incentives. This indicates that it is feasible to implement IPPCs in regular care and that the service can be managed and answered by nurses. If clinicians recognize Web-based support as effective and easy-to-access resources for their patients’ self-management support and outcomes, they might be more receptive to these types of interventions or added service options [58].

In the current study, the e-messages were primarily answered by nurses and passed on to physicians only if needed, which indicates that the IPPC can successfully be managed by nurses on the front line. Nurses are described as having a holistic approach to patients, focusing on emotional issues, consequences of disease, and illness information [59]. They are thus well equipped to answer questions and concerns and are reported to be sensitive to and able to respond to patients’ emotions expressed through e-messages [60].

WebChoice, with all its features, had added effects compared with the IPPC alone. However, development and updating of systems such as WebChoice require far more resources than an IPPC service alone. Furthermore, IPPCs can be used for different patient groups independent of diagnosis, as the patient and provider are the ones who create the content.

### Further Research

An aspect that remains to be tested is whether Web-based support systems are more effective when health care personnel with treatment responsibilities for the patients answer messages within the system, rather than health care personnel without this knowledge (patients can send e-messages anonymously). To obtain a deeper understanding of experience with the use of IPPC in routine care, we are currently interviewing nurses and physicians who have answered e-messages in this study. This experience is important to guide the implementation processes in the future. In addition, as most studies report on services between patients and physicians, more research is needed to test similar services managed by nurses. Finally, the positive effects on patients’ outcomes, despite moderate user frequencies and almost no differences detected between users and non-users, calls for further research examining how the psychological effect of simply having access to information and support might impact outcomes.

### Conclusions

In spite of the practice variations at three different hospitals, and moderate use of the IPPC service and WebChoice by study patients, our preliminary results suggest that offering Web-based support as a part of regular practice can be a powerful tool to help patients manage their illness. Our finding that the nurse-administered IPPC alone significantly reduced depression, a highly debilitating symptom, is particularly promising, as an IPPC can be implemented in different settings. However, the multicomponent support system, WebChoice, had additional positive effects on reducing anxiety symptoms and symptom distress.

## Multimedia Appendix 1

Informed consent.

## Multimedia Appendix 2

CONSORT-EHEALTH checklist V1.6.2 [32].

## Abbreviations

CBI: Cancer Behavioral Inventory
IPPC: Internet-Based Patient Provider Communication service
HADS: Hospital Anxiety and Depression Scale
LLM: linear mixed model
MSAS: Memorial Symptom Assessment Scale
RCT: randomized controlled trial
SCQ-19: Self-Administered Comorbidity Questionnaire

## References

[1] Kuijpers W, Groen WG, Aaronson NK, van Harten WH (2013). A systematic review of web-based interventions for patient empowerment and physical activity in chronic diseases: relevance for cancer survivors. J Med Internet Res.

[2] Solomon M, Wagner SL, Goes J (2012). Effects of a Web-based intervention for adults with chronic conditions on patient activation: online randomized controlled trial. J Med Internet Res.

[3] Solomon MR (2008). Information Technology to Support Self-Management in Chronic Care. Disease Management & Health Outcomes.

[4] Murray E, Burns J, See TS, Lai R, Nazareth I (2005). Interactive Health Communication Applications for people with chronic disease. Cochrane Database Syst Rev.

[5] Glasgow RE, Kurz D, King D, Dickman JM, Faber AJ, Halterman E, Woolley T, Toobert DJ, Strycker LA, Estabrooks PA, Osuna D, Ritzwoller D (2012). Twelve-month outcomes of an Internet-based diabetes self-management support program. Patient Educ Couns.

[6] Glasgow RE, Kurz D, King D, Dickman JM, Faber AJ, Halterman E, Wooley T, Toobert DJ, Strycker LA, Estabrooks PA, Osuna D, Ritzwoller D (2010). Outcomes of minimal and moderate support versions of an internet-based diabetes self-management support program. J Gen Intern Med.

[7] Lorig K, Ritter PL, Laurent DD, Plant K, Green M, Jernigan VB, Case S (2010). Online diabetes self-management program: a randomized study. Diabetes Care.

[8] Lorig KR, Ritter PL, Laurent DD, Plant K (2008). The internet-based arthritis self-management program: a one-year randomized trial for patients with arthritis or fibromyalgia. Arthritis Rheum.

[9] Powell J, Hamborg T, Stallard N, Burls A, McSorley J, Bennett K, Griffiths KM, Christensen H (2013). Effectiveness of a web-based cognitive-behavioral tool to improve mental well-being in the general population: randomized controlled trial. J Med Internet Res.

[10] van Straten A, Cuijpers P, Smits N (2008). Effectiveness of a web-based self-help intervention for symptoms of depression, anxiety, and stress: randomized controlled trial. J Med Internet Res.

[11] Stellefson M, Chaney B, Barry AE, Chavarria E, Tennant B, Walsh-Childers K, Sriram PS, Zagora J (2013). Web 2.0 chronic disease self-management for older adults: a systematic review. J Med Internet Res.

[12] Ventura F, Ohlén J, Koinberg I (2013). An integrative review of supportive e-health programs in cancer care. Eur J Oncol Nurs.

[13] Baker TB, Hawkins R, Pingree S, Roberts LJ, McDowell HE, Shaw BR, Serlin R, Dillenburg L, Swoboda CM, Han JY, Stewart JA, Carmack-Taylor CL, Salner A, Schlam TR, McTavish F, Gustafson DH (2011). Optimizing eHealth breast cancer interventions: which types of eHealth services are effective?. Transl Behav Med.

[14] Gustafson DH, McTavish FM, Stengle W, Ballard D, Hawkins R, Shaw BR, Jones E, Julèsberg K, McDowell H, Chen WC, Volrathongchai K, Landucci G (2005). Use and Impact of eHealth System by Low-income Women With Breast Cancer. J Health Commun.

[15] David N, Schlenker P, Prudlo U, Larbig W (2013). Internet-based program for coping with cancer: a randomized controlled trial with hematologic cancer patients. Psychooncology.

[16] Gustafson DH, Hawkins R, McTavish F, Pingree S, Chen WC, Volrathongchai K, Stengle W, Stewart JA, Serlin RC (2008). Internet-Based Interactive Support for Cancer Patients: Are Integrated Systems Better?. J Commun.

[17] Yun YH, Lee KS, Kim YW, Park SY, Lee ES, Noh DY, Kim S, Oh JH, Jung SY, Chung KW, Lee YJ, Jeong SY, Park KJ, Shim YM, Zo JI, Park JW, Kim YA, Shon EJ, Park S (2012). Web-based tailored education program for disease-free cancer survivors with cancer-related fatigue: a randomized controlled trial. J Clin Oncol.

[18] Ruland CM, Andersen T, Jeneson A, Moore S, Grimsbø Gh, Børøsund E, Ellison MC (2013). Effects of an internet support system to assist cancer patients in reducing symptom distress: a randomized controlled trial. Cancer Nurs.

[19] Gustafson DH, DuBenske LL, Namkoong K, Hawkins R, Chih MY, Atwood AK, Johnson R, Bhattacharya A, Carmack CL, Traynor AM, Campbell TC, Buss MK, Govindan R, Schiller JH, Cleary JF (2013). An eHealth system supporting palliative care for patients with non-small cell lung cancer: a randomized trial. Cancer.

[20] Han JY, Hawkins RP, Shaw BR, Pingree S, McTavish F, Gustafson DH (2009). Unraveling Uses and Effects of an Interactive Health Communication System. J Broadcast Electron Media.

[21] Hawkins RP, Pingree S, Baker T, Roberts LJ, Shaw B, McDowell H, Serlin R, Dillenburg L, Swoboda CM, Han JY, Stewart JA, Carmack CL, Salner A, Schlam TR, McTavish F, Gustafson DH (2011). Integrating eHealth With Human Services for Breast Cancer Patients. Transl Behav Med.

[22] Zhou YY, Kanter MH, Wang JJ, Garrido T (2010). Improved quality at Kaiser Permanente through e-mail between physicians and patients. Health Aff (Millwood).

[23] Goldzweig LC, Shekelle PG, Towfigh AA, Paige NM, Orshansky G, Haggstrom DA (2012). Systematic review: Secure messaging between providers and patients, and patients’ access to their own medical record. Evidence-based Synthesis Program.

[24] de Jong CC, Ros WJ, Schrijvers G (2014). The effects on health behavior and health outcomes of Internet-based asynchronous communication between health providers and patients with a chronic condition: a systematic review. J Med Internet Res.

[25] Wibe T, Hellesø R, Varsi C, Ruland C, Ekstedt M (2012). How does an online patient-nurse communication service meet the information needs of men with recently diagnosed testicular cancer?. ISRN Nurs.

[26] Ye J, Rust G, Fry-Johnson Y, Strothers H (2010). E-mail in patient-provider communication: a systematic review. Patient Educ Couns.

[27] McGeady D, Kujala J, Ilvonen K (2008). The impact of patient-physician web messaging on healthcare service provision. Int J Med Inform.

[28] Grimsbø Gh, Finset A, Ruland CM (2011). Left hanging in the air: experiences of living with cancer as expressed through E-mail communications with oncology nurses. Cancer Nurs.

[29] Ruland CM, Maffei RM, Børøsund E, Krahn A, Andersen T, Grimsbø Gh (2013). Evaluation of different features of an eHealth application for personalized illness management support: cancer patients' use and appraisal of usefulness. Int J Med Inform.

[30] Cornwall A, Moore S, Plant H (2008). Embracing technology: patients', family members' and nurse specialists' experience of communicating using e-mail. Eur J Oncol Nurs.

[31] Ruland CM, Jeneson A, Andersen T, Andersen R, Slaughter L, Moore SM, Bente-Schjødt-Osmo (2007). Designing tailored Internet support to assist cancer patients in illness management. AMIA Annu Symp Proc.

[32] Eysenbach G, CONSORT-EHEALTH Group (2011). CONSORT-EHEALTH: improving and standardizing evaluation reports of Web-based and mobile health interventions. J Med Internet Res.

[33] Ruland CM, Borosund E, Varsi C (2009). User requirements for a practice-integrated nurse-administered online communication service for cancer patients. Stud Health Technol Inform.

[34] Oslo University Hospital WebChoice 2.

[35] Sobin LH, Gospodarowicz M, Wittekind C, International Union Against Cancer (2010). TNM Classification of Malignant Tumours.

[36] Sangha O, Stucki G, Liang MH, Fossel AH, Katz JN (2003). The Self-Administered Comorbidity Questionnaire: a new method to assess comorbidity for clinical and health services research. Arthritis Rheum.

[37] Portenoy RK, Thaler HT, Kornblith AB, Lepore JM, Friedlander-Klar H, Kiyasu E, Sobel K, Coyle N, Kemeny N, Norton L (1994). The Memorial Symptom Assessment Scale: an instrument for the evaluation of symptom prevalence, characteristics and distress. Eur J Cancer.

[38] Hofsø K, Rustøen T, Cooper BA, Bjordal K, Miaskowski C (2013). Changes over time in occurrence, severity, and distress of common symptoms during and after radiation therapy for breast cancer. J Pain Symptom Manage.

[39] Zigmond AS, Snaith RP (1983). The hospital anxiety and depression scale. Acta Psychiatr Scand.

[40] Snaith RP (2003). The Hospital Anxiety And Depression Scale. Health Qual Life Outcomes.

[41] Bjelland I, Dahl AA, Haug TT, Neckelmann D (2002). The validity of the Hospital Anxiety and Depression Scale. An updated literature review. J Psychosom Res.

[42] Alexander S, Palmer C, Stone PC (2010). Evaluation of screening instruments for depression and anxiety in breast cancer survivors. Breast Cancer Res Treat.

[43] Merluzzi TV, Nairn RC, Hegde K, Martinez Sanchez MA, Dunn L (2001). Self-efficacy for coping with cancer: revision of the Cancer Behavior Inventory (version 2.0). Psychooncology.

[44] Kelders SM, Van Gemert-Pijnen JE, Werkman A, Nijland N, Seydel ER (2011). Effectiveness of a Web-based intervention aimed at healthy dietary and physical activity behavior: a randomized controlled trial about users and usage. J Med Internet Res.

[45] Kerr C, Murray E, Noble L, Morris R, Bottomley C, Stevenson F, Patterson D, Peacock R, Turner I, Jackson K, Nazareth I (2010). The potential of Web-based interventions for heart disease self-management: a mixed methods investigation. J Med Internet Res.

[46] Harrison JD, Young JM, Price MA, Butow PN, Solomon MJ (2009). What are the unmet supportive care needs of people with cancer? A systematic review. Support Care Cancer.

[47] Børøsund E, Cvancarova M, Ekstedt M, Moore SM, Ruland CM (2013). How user characteristics affect use patterns in web-based illness management support for patients with breast and prostate cancer. J Med Internet Res.

[48] Hinz A, Krauss O, Hauss JP, Höckel M, Kortmann RD, Stolzenburg JU, Schwarz R (2010). Anxiety and depression in cancer patients compared with the general population. Eur J Cancer Care (Engl).

[49] Burgess C, Cornelius V, Love S, Graham J, Richards M, Ramirez A (2005). Depression and anxiety in women with early breast cancer: five year observational cohort study. BMJ.

[50] Varsi C, Gammon D, Wibe T, Ruland CM (2013). Patients' reported reasons for non-use of an internet-based patient-provider communication service: qualitative interview study. J Med Internet Res.

[51] Eysenbach G (2005). The law of attrition. J Med Internet Res.

[52] Lluch M (2011). Healthcare professionals' organisational barriers to health information technologies-a literature review. Int J Med Inform.

[53] Mair FS, May C, O'Donnell C, Finch T, Sullivan F, Murray E (2012). Factors that promote or inhibit the implementation of e-health systems: an explanatory systematic review. Bull World Health Organ.

[54] André B, Inger Ringdal G, Loge JH, Rannestad T, Laerum H, Kaasa S (2008). Experiences with the Implementation of Computerized Tools in Health Care Units: A Review Article. International Journal of Human-Computer Interaction.

[55] Wakefield DS, Mehr D, Keplinger L, Canfield S, Gopidi R, Wakefield BJ, Koopman RJ, Belden JL, Kruse R, Kochendorfer KM (2010). Issues and questions to consider in implementing secure electronic patient-provider web portal communications systems. Int J Med Inform.

[56] Nijland N, van Gemert-Pijnen J, Boer H, Steehouder MF, Seydel ER (2008). Evaluation of internet-based technology for supporting self-care: problems encountered by patients and caregivers when using self-care applications. J Med Internet Res.

[57] Singh H, Fox SA, Petersen NJ, Shethia A, Street RL (2009). Older patients' enthusiasm to use electronic mail to communicate with their physicians: cross-sectional survey. J Med Internet Res.

[58] Curry SJ (2007). eHealth research and healthcare delivery beyond intervention effectiveness. Am J Prev Med.

[59] Patel VL, Cytryn KN, Shortliffe EH, Safran C (2000). The collaborative health care team: the role of individual and group expertise. Teach Learn Med.

[60] Grimsbø Gh, Ruland CM, Finset A (2012). Cancer patients' expressions of emotional cues and concerns and oncology nurses' responses, in an online patient-nurse communication service. Patient Educ Couns.

