# Supplementary material for: Comparing Effects in Regular Practice of E-Communication and Web-Based Self-Management Support Among Breast Cancer Patients: Preliminary Results From a Randomized Controlled Trial
Source: J Med Internet Res. 2014 Dec 18;16(12):e295. doi: 10.2196/jmir.3348 (PMC4285721; doi:10.2196/jmir.3348)
Supplement: Supplementary file 1 [file jmir_v16i12e295_app1.pdf]

Forespørsel om deltakelse i forskningsprosjektet
Effekt av internettstøtte for kreftpasienter som en del av klinisk praksis (WebChoice 2.0).

Bakgrunn og hensikt
Dette er en forespørsel til deg om å delta i et forskningsprosjekt for å teste ut effekten av et internettbasert støtteprogram som er utviklet for mennesker med kreft (WebChoice 2.0). Tilbudet er utviklet for støtte mellom og etter opphold ved sykehuset. WebChoice 2.0 har flere komponenter: mulighet til å nedtegne de problemer/plager man har, database med tiltak man iverksette mot disse plagene i hjemmesituasjonen, kvalitetssikrede internettlinker til informasjon om sykdom og behandling, diskusjonsforum med andre pasienter i samme situasjon, samt mulighet til å stille spørsmål via e-post i et sikkert system (spørsmål- og svartjeneste) til sykepleier ved sykehuset. Ved behov kan spørsmål videreformidles til lege ved sykehuset eller rådgiver hos Helseøkonomiforvaltningen (HELFO). Vi ønsker å undersøke om denne tjenesten eller deler av den kan hjelpe pasienter til å håndtere sykdommen og mestre hverdagen bedre. Vi vil også undersøke om bruk av tjenesten kan ha betydning for utgifter knyttet til sykdommen, samt behov for helse- og sosialtjenester fra det offentlige.

Studien utgår fra Oslo universitetssykehus HF, Rikshospitalet. Du forespørres om å delta i studien fordi du er pasient ved et av våre samarbeidende sykehus.

For å undersøke om denne internettjenesten er nyttig, vil studiedeltakerne bli inndelt i tre grupper; én gruppe som får tilgang til spørsmål- og svartjenesten, én gruppe som får tilgang til alle komponentene i WebChoice 2.0 og én sammenligningsgruppe som får det vanlige tjenestetilbudet fra sykehuset, uten disse spesielle tjenestene. Hvis du sier ja til å delta i studien, vil du bli tilfeldig trukket ut til å inngå i én av gruppene.

Hva innebærer studien?
Deltagelse i studien går over 18 måneder, hvorav bare de første 12 månedene fordrer noen aktivitet fra din side. Dersom du blir trukket ut til å være med i gruppen som får tilbud om å bruke spørsmål- og svartjenesten eller hele WebChoice 2.0, innebærer det at du i 12 måneder vil kunne benytte deg av tjenesten så mye du ønsker. Som bruker av tjenesten logger du deg på slik du logger deg på din nettbank. Dette vil du få nærmere forklaring på. All informasjon som utveksles er beskyttet gjennom strenge datatekniske sikkerhetstiltak og vil bli kryptert og liggende i et sikkert system ved Oslo universitetssykehus HF, Rikshospitalet.
Enten du kommer i den gruppen som får tilgang til spørsmål- og svartjenesten, WebChoice 2.0 eller sammenligningsgruppen, vil vi be deg fylle ut noen opplysninger om deg selv på spørreskjemaer ved oppstart. Vi vil så sende deg spørreskjemaer etter 2, 4, 6, 8 og 12 måneder, som inneholder spørsmål om hvordan du har det i forbindelse med sykdom og behandling. Disse vil du bli bedt om å returnere til oss i en vedlagt ferdigfrankert konvolutt. Det vil ta ca ½ time å fylle ut skjemaene. Dersom du kommer i den gruppen som får tilbud om å bruke WebChoice 2.0 eller spørsmål- og svartjenesten vil du tillegg motta spørreskjema som omhandler hvor nyttig og brukervennlig du opplevde tjenestene.

I tillegg til data som samles inn gjennom spørreskjema ber vi om din tillatelse til å innhente følgende:

- Data for hvordan du benytter tjenestene (hva som benyttes, hvor ofte, hvor lenge, innhold i meldinger, notater og kommunikasjon med andre pasienter).
- Opplysninger fra offentlige registre (se utdyping under avsnittet om personvern) 12 og 18 måneder etter din oppstart i studien om offentlige ytelser forbundet med sykdommen og bruk av helse- og sosialtjenester, evt. sykehusinnleggelser i aktuelle periode, samt ytelser til reseptbelagte legemidler.
- Enkelte opplysninger om nåværende sykdom og behandling fra din journal ved sykehuset.

Om du ikke ønsker å delta i denne studien, vil du motta vanlig behandlingstilbud ved den avdelingen du behandles ved.

Mulige fordeler og ulemper
Studien medfører ingen kostnader for deg og det er ingen risiko forbundet med studien. Gjennom din deltakelse vil du, enten du deltar i en av de to gruppene som prøver ut tjenestene eller i sammenligningsgruppen, bidra til viktig kunnskap om hvordan et tilpasset program for internettstøtte kan være til hjelp for mennesker med alvorlig sykdom.

Fordeler for deg, dersom du blir trukket ut til å være med i den gruppen som får tilgang til spørsmål- og svartjenesten, vil være at du får mulighet til å benytte tjenesten etter og mellom sykehusopphold. Du kan stille spørsmål via elektroniske meldinger og få råd og veiledning fra sykepleier, og ved behov kan dine spørsmål videreformidles til lege ved sykehuset eller rådgiver ved HELFO. De som besvarer meldingene fra deg har spesialkunnskap om din sykdom og behandling. Rådgivere ved HELFO vil få videreformidlet aktuelle spørsmål i anonymisert form fra sykepleier som betjener spørsmål- og svartjenesten. Dersom du blir trukket ut til gruppen som får tilgang til WebChoice 2.0 vil du i tillegg få tilgang til kvalitetssikret informasjon om tiltak som du kan iverksette selv mot sykdomsrelaterte plager. Du vil også få mulighet til å registrere egne plager, for eksempel som forberedelse til legebesøk, og mulighet til å kommunisere med mennesker i samme situasjon. Å kunne stille spørsmål og få svar fra fagpersoner uansett hvor du oppholder deg, samt ha tilgang til kvalitetssikret informasjon om sykdommen kan kanskje hjelpe deg å håndtere sykdommen og eventuelle komplikasjoner bedre når du er hjemme.

Det er få ulemper og ubehag knyttet til deltakelse i studien. Noen vil kanskje oppleve det som slitsomt å svare på spørreskjemaer.

Hva skjer med informasjonen om deg?
Informasjonen som registreres om deg skal kun brukes slik som beskrevet i hensikten med studien. Alle opplysningene vil bli behandlet uten navn og fødselsnummer eller andre direkte gjenkjennende opplysninger. En kode (studie-ID) knytter deg til dine opplysninger gjennom en navneliste. Navnelisten er atskilt fra alle opplysninger vi samler om studiedeltakerne. Det er kun autorisert personell knyttet til prosjektet som har adgang til navnelisten og som kan finne tilbake til deg. Hvis det kommer frem noe i korrespondansen i spørsmål- og svartjenesten som er viktig for din behandling ved sykehuset, vil dette bli dokumentert i pasientjournalen.
Det er innhentet nødvendig konsesjon fra Datatilsynet for å kunne sammenstille opplysninger fra NAV, HELFO, Norsk Pasientregister og Reseptregisterets databaser med studieopplysninger. All informasjon om deg vil slettes etter at studien er avsluttet, senest 31.12.2025.
Det vil ikke være mulig å identifisere deg i resultatene av studien når disse publiseres. Studien er godkjent av Regional Etisk Komité (REK) Sør-Øst, Protokollutvalget og Personvernombudet ved Oslo universitetssykehus HF, Rikshospitalet.

Frivillig deltakelse
Det er frivillig å delta i studien. Du kan når som helst og uten å oppgi noen grunn trekke ditt samtykke til å delta i studien. Dette vil ikke få konsekvenser for din videre behandling. Dersom du ønsker å delta, undertegner du samtykkeerklæringen på siste side. Om du nå sier ja til å delta, kan du senere trekke deg fra studien uten at det påvirker din øvrige behandling. Du kan i så fall også be om at de opplysninger vi allerede har fått fra deg blir slettet.

Dersom du har spørsmål om studien, kan du kontakte prosjektleder Cornelia Ruland, tlf 23 07 54 60, stipendiat Elin Børøsund, tlf 23 07 54 52 eller enhetsleder ved Pasienthotellet, Sørlandet sykehus, Kristiansand, Ellen B. Mjøs, 38 12 53 40.

Kapittel A- utdypende forklaring av hva studien innebærer

Bakgrunnsinformasjon om studien:
Mennesker med alvorlig sykdom kan oppleve mange problemer og bekymringer. Når de er hjemme mellom eller etter behandling er det ofte begrenset tilgang til profesjonell hjelp. Internettbaserte tjenester har vist seg å være nyttige i forhold til å støtte pasienter til å mestre daglige utfordringer og behov. Derfor vil vi undersøke i hvilken grad WebChoice 2.0 eller kun en spørsmål– og svartjeneste kan hjelpe pasienter i en slik situasjon, med tanke på å ha det bedre gjennom sykdom og behandling, med mindre symptomer/plager og bekymringer, og bedre livskvalitet. Når en kan få hjelp umiddelbart når problemer oppstår, kan dette kanskje også bidra til raskere rehabilitering, forhindre komplikasjoner, styrke egenkompetanse, redusere medikamentbruk og å kunne komme raskere tilbake i arbeid.

Hvis denne studien viser at det er nyttig for deltakerne, vil det i framtiden være aktuelt å utvikle tilsvarende tjenester som kanskje kan bli en del av det ordinære tjenestetilbudet til pasienter med alvorlig sykdom.

Kriterier for å delta i studien er at du er over 18 år, behersker norsk skriftlig og muntlig, har tilgang til internett og bruker nettbank med BankID som påloggingsnøkkel.

Kapittel B - Personvern, økonomi og forsikring

Personvern
Data som vil bli registrert om deg den tiden du deltar i studien er:

- opplysninger innhentet gjennom spørreskjema
- kommunikasjon med helsepersonell i spørsmål- og svartjenesten
- bruk av spørsmål- og svartjenesten og de ulike delene i WebChoice 2.0 (fra systemlogg)
- opplysninger om nåværende sykdom og behandling fra pasientjournalen
- sykefravær og ytelser til, rehabilitering, attføring eller uføretrygd (fra NAV)
- besøk hos fastlege, spesialist, fysioterapeut eller bruk av dietetiske næringsmidler (fra HELFO)
- sykehusopphold i form av poliklinikkbesøk eller innleggelser (Norsk Pasientregister)
- utgifter til legemidler relatert til smerter, angst, depresjon og søvn (Reseptregisteret)

Det foreligger konsesjon fra Regional etisk komité for dette og databehandlerkontrakt vil bli utarbeidet før innhenting av data fra eksterne registre. Opplysningene fra NAV, HELFO og Norsk Pasientregister innhentes fra deres respektive databaser av personer som har tjenestemessig tilgang til disse databasene. De vil få tilsendt studiedeltakernes personnummer og deres studie-ID fra oss på en CD-

ROM og henter ut de aktuelle opplysningene om studiedeltakerne fra sin database via personnummeret. Når opplysningene returneres til oss på CD-ROM (enten med kurer eller som rekommandert sending), vil personnummer være slettet, og kun studie-ID knytter opplysningene til hver enkelt studiedeltaker. Ved ankomst til navngitt medlem av forskningsteamet lagres data umiddelbart på sikker server for forskning ved sykehuset, og CD-ROM destrueres.
Norsk Pasientregister forvaltes av Helsedirektoratet og Reseptregisteret av Folkehelseinstituttet. Ved Reseptregisteret vil data bli innhentet ved at vi på tilsvarende måte overfører filer med personnummer og opplysninger fra studien som skal ses opp mot data fra Reseptregisteret. Filene vil bli videresendt til Statistisk sentralbyrå (SSB) for avidentifisering og påføring av pseudonyme personnummer tilsvarende de som brukes i Reseptregisteret. Når vi mottar datafilene fra Reseptregisteret og lagrer dem på vår sikre forskningsserver, vil dataene være knyttet til pseudonymnummer. Dataene fra Reseptregisteret vil på denne måten ikke kunne spores til hver enkelt studiedeltaker, men være fullstendig anonymiserte.
Kun navngitte medlemmer av forskningsteamet vil der ha tilgang til dataene. De vil ikke være tilgjengelige for personell som kommuniserer med pasientene i WebChoice 2.0. Alle medlemmene av forskningsteamet har taushetsplikt.
Oslo universitetssykehus HF, Rikshospitalet ved administrerende direktør er databehandlingsansvarlig.

Rett til innsyn og sletting av opplysninger om deg
Hvis du sier ja til å delta i studien, har du rett til å få innsyn i hvilke opplysninger som er registrert om deg. Du har videre rett til å få korrigert eventuelle feil i de opplysningene vi har registrert. Dersom du trekker deg fra studien, kan du kreve å få slettet innsamlede opplysninger, med mindre opplysningene allerede er inngått i analyser eller brukt i vitenskapelige publikasjoner.

Økonomi og Helse Sør-Østs rolle
Studien er finansiert gjennom forskningsmidler fra Helse Sør-Øst, og bekostes også av Oslo Universitetssykehus HF, Rikshospitalet, Universitetssykehuset Nord-Norge HF - Tromsø, Sykehuset Vestfold HF - Tønsberg og Sørlandet sykehus HF - Kristiansand. Det er ingen interessekonflikter å melde.

Forsikring
Du er forsikret på samme måte som ved ordinære opphold/konsultasjoner ved sykehuset.

Informasjon om utfallet av studien
Som deltaker i studien har du rett til å få informasjon om utfallet/resultatet av studien.

WebChoice 2.0

Reg. Nr: | | | |

Initialer: | | | |

Samtykke til deltakelse i studien

"Effekt av internettstøtte for kreftpasienter som en del av klinisk praksis" (WebChoice 2.0)

Jeg er villig til å delta i studien:

---(navn i blokkbokstaver)

(Signert av prosjektdeltaker, dato)

Jeg bekrefter å ha gitt informasjon om studien

(Signert, rolle i studien, dato)
