# Supplementary material for: Comparing Effects in Regular Practice of E-Communication and Web-Based Self-Management Support Among Breast Cancer Patients: Preliminary Results From a Randomized Controlled Trial
Source: J Med Internet Res. 2014 Dec 18;16(12):e295. doi: 10.2196/jmir.3348 (PMC4285721; doi:10.2196/jmir.3348)
Supplement: Supplementary file 2 [file jmir_v16i12e295_app2.pdf]

CONSORT-EHEALTH Checklist V1.6.2 Report

(based on CONSORT-EHEALTH V1.6), available at [http://tinyurl.com/consort-ehealth-v1-6].

3348

Date completed

8/29/2014 7:59:19

by

Elin Børøsund

Comparing Effects in Regular Practice of E-communication and Web-Based Self-Management Support on Symptom Distress, Anxiety, Depression and Self-Efficacy among Breast Cancer Patients. Preliminary Results from a Randomized Controlled Trial

TITLE

1a-i) Identify the mode of delivery in the title

"Comparing Effects in regular Practice of E-communication and Web-Based Self-Management Support..."

1a-ii) Non-web-based components or important co-interventions in title

This is not relevant as both interventions are Web-based

1a-iii) Primary condition or target group in the title

Breast cancer patients

ABSTRACT

1b-i) Key features/functionalities/components of the intervention and comparator in the METHODS section of the ABSTRACT

"The nurse-administered IPPC allowed patients to send secure e-messages to and receive e-messages from health care personnel at the hospital at which they were treated. In addition to the IPPC, WebChoice contains components for symptom monitoring, tailored information and self-management support, a diary, and communication with other patients."

1b-ii) Level of human involvement in the METHODS section of the ABSTRACT

"Twenty care providers (11 nurses, 6 physicians and 3 social workers) were trained to answer questions from patients."

1b-iii) Open vs. closed, web-based (self-assessment) vs. face-to-face assessments in the METHODS section of the ABSTRACT

"167 patients recently diagnosed with breast cancer and undergoing treatment were recruited from three Norwegian hospitals."
"Outcomes were measured with questionnaires at study entry, and at study months 2, 4 and 6."

1b-iv) RESULTS section in abstract must contain use data

"Patients were randomly assigned to the WebChoice group (n=64), the IPPC group (n=45), or the usual care group (n=58). Response rates to questionnaires were 73.7% (123/167) at two months, 65.9 (110/167) at four months, and 62.3% (104/167) at six months. Attrition was similar in all study groups. Among those with access to WebChoice, 64% (41/64) logged on more than once and 39% (25/64) sent e-messages to care providers. In the IPPC group, 40% (18/45) sent e-messages. Linear mixed models analyses revealed that the WebChoice group reported significantly lower symptom distress (Mean diff: .16, 95% CI: .06-.25, P=.001), anxiety (Mean diff: .79, 95% CI: .09-1.49, P=.03) and depression (Mean diff: .79, 95% CI: .09-1.49, P=.03) compared with the usual care group. The IPPC group reported significant lower depression scores compared with the usual care group (Mean diff: .69, 95% CI: .05-.1.32, P=.03), but no differences were observed for symptom distress or anxiety. No significant differences in self-efficacy were found among the study groups."

1b-v) CONCLUSIONS/DISCUSSION in abstract for negative trials

The trial showed positive impact from the interventions

INTRODUCTION

2a-i) Problem and the type of system/solution

"…,Web-based support systems often consist of several components that make it difficult to determine which components are most beneficial for patients, and little is known about the relative contribution of different components."
"Thus, we know little about the effect of stand-alone IPPCs and patient outcomes and how they compare to more comprehensive Web-based support systems where IPPCs are one of several components."
"WebChoice is a Web-based illness management support system based on patient-centered principles and designed to support cancer patients in self-management of their illness, independent of location and time."
"As several studies show benefits of Web-based support systems, it is timely to examine the relative contribution of different components of these multi-component support systems aiming to improve selected outcomes……… Furthermore, there is a need to test the effects of Web-based support as a part of regular care. "

2a-ii) Scientific background, rationale: What is known about the (type of) system

"… such interventions have been shown to assist a wide range of patients [1-11]. In cancer care, Web-based interventions are described as helpful for individuals [12]. This includes findings of increased health information competence [13,14], emotional processing [13], fighting spirit [15], social support [14,16], quality of life [16,17], as well as reductions in symptom distress [18,19], and decrease in depression and anxiety scores [17].
However, Web-based support systems often consist of several components that make it difficult to determine which components are most beneficial for patients, and little is known about the relative contribution of different components [13]." "Knowledge of the use and effects of single components on patient outcomes will be important to determine component candidates for inclusion in Web-based support systems [13,16,21].
"Several studies report benefits from using Internet-based patient-provider communication services (IPPC) for communication between patients and health care providers in terms of assisting patients in managing illness and improving health outcomes [22-24], addressing unmet communication needs [25,26], increasing satisfaction [23,27], and improving quality of care [22,27].." "The IPPC was rated by patients as the most valuable component of WebChoice [28].
"High levels of satisfaction with a nurse-administered IPPC were also reported in a study by Cornwall et al [30], but effects on patients' outcomes of IPPCs alone are rarely described. Thus, we know little about the effect of stand-alone IPPCs and patient outcomes and how they compare to more comprehensive Web-based support systems where IPPCs are one of several components."
"Results from a previously randomized clinical trial (RCT) that followed 325 breast- and prostate-cancer patients for one year showed that patients with access to WebChoice had significantly reduced symptom distress compared with the usual care control group [18]." "One of the WebChoice features most valued by the study participants, as reported in the previous study, was the opportunity to send e-messages to expert nurses in cancer care, who responded to patients' questions and concerns within 24 hours [29]."

METHODS

3a) CONSORT: Description of trial design (such as parallel, factorial) including allocation ratio

" The aims of the study were therefore to test and compare the effects of (1) an IPPC, (2) the multi component WebChoice intervention (including an IPPC), and (3) usual care (control group) on symptom distress, anxiety, and depression (primary outcomes), as well as self-efficacy (secondary outcome) after 6 months of follow-up. In addition, explorative sub-analyses were performed to detect whether the outcomes were associated with the actual use of the interventions. We hypothesized that; the WebChoice group compared with the usual care group, would have better outcomes than the IPPC group compared with the usual care group. We also hypothesized that both groups would have better outcomes than the usual care group, on both primary and secondary outcomes."

3b) CONSORT: Important changes to methods after trial commencement (such as eligibility criteria), with reasons
No changes
3b-i) Bug fixes, Downtimes, Content Changes
"The system experienced nine day period of downtime during the first year due to technical problems at the hospital server that hosted the application. No changes were made to the interventions during the trial period apart from fixing minor bugs."
4a) CONSORT: Eligibility criteria for participants
"Inclusion criteria were recent diagnosis of breast cancer treated with surgery, or under treatment with radiation, chemotherapy, hormone therapy, or combinations of those (maximum 12 months after surgery), age over 18 years, able to write/read and speak Norwegian, having access to the Internet at home and with a public key solution for secure system access (PKI)."
4a-i) Computer / Internet literacy
Computer/Internet literacy was not a criteria for eligibility and is therefore not addressed in the method section
Internet access was however a criterion: "…having access to Internet at home and with a public key solution for secure system access (PKI)."
Computer literacy See comment 4a
4a-ii) Open vs. closed, web-based vs. face-to-face assessments:
"Eligible patients scheduled for surgery or coming for check-ups after surgery or treatment were identified by the study nurses at the hospitals and provided with information about the study."
4a-iii) Information giving during recruitment
"Eligible patients scheduled for surgery or coming for check-ups after surgery or treatment were identified by the study nurses at the hospitals and provided with information about the study. Upon patients' arrival at the clinic, the study nurses met the patients, provided brief information about the study and asked if they were interested in participating. If the patients agreed, the nurse informed them about the study's purpose and procedures, and asked for written informed consent."

After randomization, all patients were informed by the study nurse of their group assignment.
"Patients who were randomized into the IPPC or WebChoice groups were informed and instructed in the use of the IPPC or WebChoice."
"They received a printed user manual with instructions for use, how to log on to the system, contact address and phone number for help if needed. The only in-person information given was instructions on how to access the site and how to connect with the study support service if questions occurred. The study nurses showed them where in the user manual they could to find information about how to access the site and how to connect with the study support service if needed. In addition the participants were informed that they could use the IPPC or any component of WebChoice as much or as little as they liked, and that using the system was entirely voluntary."
4b) CONSORT: Settings and locations where the data were collected

"Consenting patients completed baseline questionnaires before randomization."
"All participants were sent questionnaires by postal mail at 2, 4 and 6 months after enrolling."
4b-i) Report if outcomes were (self-)assessed through online questionnaires
No online questionnaires, see 4b.
4b-ii) Report how institutional affiliations are displayed
The information about the study included logos and contact information for all participatory hospitals. The website had the logo of Oslo University hospital, but this was not reported in the manuscript as we do not believe this impacted the results.
5) CONSORT: Describe the interventions for each group with sufficient details to allow replication, including how and when they were actually administered
5-i) Mention names, credential, affiliations of the developers, sponsors, and owners
The interventions were developed at Oslo University Hospital
"Conflict of interests: The last author (CR) is the developer of WebChoice, but has no financial or ownership rights to the application."
5-ii) Describe the history/development process
"The IPPC is a further development of the IPPC component described in a previous study of WebChoice"
"WebChoice was developed in close cooperation with users and health care personnel.
After testing the system in a RCT, the system was refined based on responses from users through questionnaires and focus groups. In addition a blog feature was included. The WebChoice version tested in the current study targeted breast cancer patients, and contained in addition to the IPPC service, the following components (Figure 2):"
5-iii) Revisions and updating
"The advice component was updated once a year."
"External links were automatically checked every fourth week to ensure that they were still active."
"No changes were made to the interventions during the trial period apart from fixing minor bugs."
5-iv) Quality assurance methods
"The IPPC is a further development of the IPPC component described in a previous study of WebChoice"
"WebChoice was developed in close cooperation with users and health care personnel.
After testing the system in a RCT, the system was refined based on responses from users through questionnaires and focus groups. In addition a blog feature was included
5-v) Ensure replicability by publishing the source code, and/or providing screenshots/screen-capture video, and/or providing flowcharts of the algorithms used
We have provided a screenshot of the front page of WebChoice in the manuscript (figure 2)
The components in WebChoice are described in the manuscript and the flow of messages between patients and health care providers is displayed in figure 1.
5-vi) Digital preservation
The interventions are only accessible with a user name and a password.
We have provided a screenshot of the front page of WebChoice in the manuscript (figure 2)
5-vii) Access

"After being informed about group assignment, the patients were given access to the interventions the same day. They received an automatic welcome message when the system was ready to use. There was an option to be notified by SMS or regular e-mail when new messages appeared in the system."

The participants accessed the applications from their home. They did not pay anything to participate/to use the applications and did not get reimbursed for participation.

Health care providers accessed the application during regular working hours: "There was a clear schedule for who was responsible for answering patients' messages. The nurses were frontline, and received all messages first. If necessary, they could forward the message to other care providers. . If considered important, information from e-messages could be copied into the medical record and made available for other health care providers. When new questions arrived in the system, the recipient was notified through the hospital's e-mail system or by SMS. The same providers answered e-messages from both the IPPC and WebChoice groups using the same interface."

5-viii) Mode of delivery, features/functionalities/components of the intervention and comparator, and the theoretical framework

The IPPC: "It allows patients to seek help from health care personnel at their treatment hospital. They can ask questions, share experiences with or get advice from oncology nurses. If needed, the nurse can pass on their question to physicians and social workers (Figure 1). The system has a high security level, where both patients and health care providers log into the system with strong authentication keys. Care providers had access to the patients' medical record at the hospital. The patient questions were asynchronous and were answered within two workdays, usually within one day"

WebChoice: "The WebChoice version tested in the current study targeted breast cancer patients, and contained the following components in addition to the IPPC service (figure 2):

1.An assessment component where patients could monitor their symptoms, problems and priorities for support along physical, functional and psychosocial dimensions. From a predefined list, patients could choose symptoms and problems they were experiencing, rate the burden of these and indicate where they needed help. This information could be used to monitor improvement/deterioration of the condition; indicate when to alert health care personnel; prepare for a hospital/physician consultation; improve patient-provider communication; or to obtain immediate access to the self-management advice components described below.

2.An advice component provided illness self-management support. The patients' self-reported symptoms triggered the display of appropriate self-management activities that patients could choose from to relieve symptoms and problems. The component could also be used without finishing an assessment first. Each choice contained an explanation of what the activity was, how to perform it, potential risks, side effects, contraindications, when to contact a physician, levels of evidence, references to the source of the evidence, and links to other reliable websites for related information. The advice component was updated once a year.

3.An information component where patients had access to other reliable Web sources in Norwegian and English, such as information about tests, treatments and potential side effects, lifestyle suggestions, and information about patients' legal rights. External links were automatically checked every fourth week to ensure that they were still active.

4.A communication component for sharing experiences with other patients. Patients could participate in an online forum group discussion that allowed them to exchange messages anonymously with other patients, or use a blog. The forum and blog were monitored by nurses at our research center. The nurses did not participate in the forum or blog, but answered two postings in the forum that were not answered by the other participants.

5.An electronic diary where patients could keep personal notes."

5-ix) Describe use parameters

"Participants were told that they could use the IPPC or any component of WebChoice as much or as little as they liked, and that using the system was entirely voluntary."

5-x) Clarify the level of human involvement

"Care providers (n=20; 11 nurses, 6 physicians and 3 social workers) who answered questions from patients consisted of a dedicated group of expert nurses and physicians in breast cancer care, and social workers at the hospital where the patients were treated." "The nurses were frontline, and received all messages first. If necessary, they could forward the message to other care providers."

"The same providers answered e-messages from both the IPPC and WebChoice groups using the same interface…….The health care providers had no access to details about how patients used other components of WebChoice."

"The forum and blog were monitored by nurses at our research center. The nurses did not participate in the forum or blog, but answered two postings in the forum that were not answered by the other participants."

5-xi) Report any prompts/reminders used

There were no prompts to use the interventions.

5-xii) Describe any co-interventions (incl. training/support)

No co-intervention

6a) CONSORT: Completely defined pre-specified primary and secondary outcome measures, including how and when they were assessed

"The primary outcomes were symptom distress, anxiety and depression. The secondary outcome was self-efficacy. All outcomes were measured at baseline, 2, 4 and 6 months thorough self-assessed questionnaires sent to participants by postal mail."

6a-i) Online questionnaires: describe if they were validated for online use and apply CHERRIES items to describe how the questionnaires were designed/deployed

No online questions used

6a-ii) Describe whether and how "use" (including intensity of use/dosage) was defined/measured/monitored

"Data on system use were extracted from the user logs on the server. Information was collected on how many times the users had logged on and which components of WebChoice were accessed or used actively."

6a-iii) Describe whether, how, and when qualitative feedback from participants was obtained

Feedback about participant experiences from intervention use was collected at eight months. These results are not reported in the current manuscript and will be part of another publication.

6b) CONSORT: Any changes to trial outcomes after the trial commenced, with reasons

No changes after trial commenced

7a) CONSORT: How sample size was determined

7a-i) Describe whether and how expected attrition was taken into account when calculating the sample size

In the study protocol, we used statistical power of 80 % to detect an effect size of 0.25 at the 5% significance level and a sample size of 369 respondents was originally calculated. This sample accounted for an expected 30 % dropout rate during the study period and a 70 % utilization rate of the intervention, similar to observations in a previous RCT og WebChoice (Ruland 2013). Due to slower recruitment than anticipated we chose to stop study inclusion after 200 consenting participants.

As the study failed to reach the calculated sample, we chose to not present sample size calculation in the manuscript. As can be seen in the paper, the final sample size was sufficient to reveal statistically sign differences as the effects sizes were larger than originally anticipated.

7b) CONSORT: When applicable, explanation of any interim analyses and stopping guidelines

The decision to analyze 6 month data on a subsample was based on a prolonged recruitment of the last respondents in the study. We chose to present interim 6 months results to avoid delayed publication of the results.
There were no stopping guidelines of the study

8a) CONSORT: Method used to generate the random allocation sequence

“After completion of baseline questionnaires, patients were randomized according to a pre-defined automated computerized block randomization, with a block size of 42 stratified by site”

8b) CONSORT: Type of randomisation; details of any restriction (such as blocking and block size)

“After completion of baseline questionnaires patients were randomized according to a pre-defined automated computerized block randomization, with a block size of 42 stratified by site.”

9) CONSORT: Mechanism used to implement the random allocation sequence (such as sequentially numbered containers), describing any steps taken to conceal the sequence until interventions were assigned

The randomization was performed by a research support center, independent of researchers as well as study nurses.

10) CONSORT: Who generated the random allocation sequence, who enrolled participants, and who assigned participants to interventions

An independent research support center performed the randomization.
“Eligible patients scheduled for surgery or coming for check-ups after surgery or treatment were identified by the study nurses at the hospitals and provided with information about the study. Upon patients’ arrival at the clinic, the study nurses met the patients, provided brief information about the study and asked if they were interested in participating. If the patients agreed, the nurse informed them about the study’s purpose and procedures, and asked for written informed consent.”
“Patients who were randomized into the IPPC or WebChoice groups were informed and instructed in the use of the IPPC or WebChoice“, by the study nurse at the hospital.
The first author gave access to the interventions.

11a) CONSORT: Blinding - If done, who was blinded after assignment to interventions (for example, participants, care providers, those assessing outcomes) and how

11a-i) Specify who was blinded, and who wasn’t

“Due to the content of the interventions, patients could not be blinded to which arm they were randomized.”
“The same providers answered e-messages from both the IPPC and WebChoice groups using the same interface. However, they were not entirely blinded to the intervention group assignment, because this was sometimes disclosed by patients through the messages.”
The researchers were not blinded to which groups the patients were assigned to.

11a-ii) Discuss e.g., whether participants knew which intervention was the “intervention of interest” and which one was the “comparator”

“Due to the content of the interventions, patients could not be blinded to which arm they were randomized.”
They knew from the study information which different groups patients could be assigned to.

11b) CONSORT: If relevant, description of the similarity of interventions

Patients in the WebChoice group had access to the same IPPC service as the IPPC group. In addition, they had access to the following components: An assessment component to monitor symptoms, an advice component with illness self-management support, an information component with access to other reliable Web sources, and a communication component for sharing experiences with other patients in a forum or a blog and a diary.

12a) CONSORT: Statistical methods used to compare groups for primary and secondary outcomes

“For analysis of between group differences in symptom distress, anxiety and depression (primary outcomes) and self-efficacy (secondary outcome), linear mixed models (LMM) for repeated measures were fitted. A diagonal covariance structure was used to model dependencies among measurements on the same individual at different time points. Models for each outcome consisted of 3 effects: measurement occasion (time), interventions (WebChoice, IPPC, usual care) and the interaction of time and intervention. All measured time points of the outcome variables are considered and the LMM approach therefore adjusts for baseline differences. To test whether potential confounders impacted the results, LMM adjusted for variables such as site, age, marital status, education, time since diagnosis, stage of disease and comorbidity were fitted. Compared to the unadjusted models, these adjusted models revealed even larger differences in favor of the intervention groups compared to the usual care group. Taking the limited sample size into account and aiming to avoid over fitting, only the results from the unadjusted models are presented. As no statistically significant differences were observed between the study groups on demographic and disease related factors at baseline, these models were not further adjusted for the possible confounders. The authors are aware that this might underestimate the true differences between the groups. Analyses of primary and secondary outcomes were conducted on an intention-to-treat basis, including all participants in each group, independent of whether they were users or non-users of the interventions. “

12a-i) Imputation techniques to deal with attrition / missing values

“No imputation of missing data was necessary or performed, as the LMM uses all data available to estimate the covariance matrix and model the dependencies.”

12b) CONSORT: Methods for additional analyses, such as subgroup analyses and adjusted analyses

“In addition, explorative sub-analyses were performed to detect whether the outcomes were associated with the actual use of the interventions. LMM for repeated measures were fitted. Models for each outcome were fitted with three factors: measurement occasion (time), interventions (user/non- user of WebChoice and IPPC) and the interaction of time and intervention. Age was added as a covariate because age is known to be associated with willingness to use Web-based tools[43,44].”

RESULTS

13a) CONSORT: For each group, the numbers of participants who were randomly assigned, received intended treatment, and were analysed for the primary outcome

Yes, see figure 3 (flowchart)

13b) CONSORT: For each group, losses and exclusions after randomisation, together with reasons

Yes, see figure 3 (flowchart)

13b-i) Attrition diagram

No, the participants have access to the system up to 12 months post intervention.
See table 3 for logons and usage of different components.

14a) CONSORT: Dates defining the periods of recruitment and follow-up

“These participants were recruited between May 2010 and September 2012.”

14a-i) Indicate if critical “secular events” fell into the study period

No secular events occurred during the study period.

14b) CONSORT: Why the trial ended or was stopped (early)

“Due to a slower recruitment than anticipated we had to stop inclusion after 200 consenting participants,..”

15) CONSORT: A table showing baseline demographic and clinical characteristics for each group

Yes, see table 1

15-i) Report demographics associated with digital divide issues

Yes, see table 1

16a) CONSORT: For each group, number of participants (denominator) included in each analysis and whether the analysis was by original assigned groups
16-i) Report multiple "denominators" and provide definitions
Yes, see table 1, 2 and 3 in the manuscript
16-ii) Primary analysis should be intent-to-treat
"Analyses of primary and secondary outcomes were conducted on an intention-to-treat basis, including all participants in each group, independent of whether they were users or non-users of the interventions. the level of use of the interventions." Results are shown in figure 4 and table 2.
17a) CONSORT: For each primary and secondary outcome, results for each group, and the estimated effect size and its precision (such as 95% confidence interval)
Yes, these results are reported in table 2
17a-i) Presentation of process outcomes such as metrics of use and intensity of use
Yes, we report on logons to the two interventions as well as logons/use of the different components. See table 3.
17b) CONSORT: For binary outcomes, presentation of both absolute and relative effect sizes is recommended
No binary outcomes.
18) CONSORT: Results of any other analyses performed, including subgroup analyses and adjusted analyses, distinguishing pre-specified from exploratory
"In addition, explorative sub-analyses were performed to detect whether the outcomes were associated with the actual use of the interventions."
18-i) Subgroup analysis of comparing only users
The only exploratory analysis we present is our analysis of use and relations to outcome
19) CONSORT: All important harms or unintended effects in each group
No adverse events were detected during the 6 months of the study.
19-i) Include privacy breaches, technical problems
No privacy breaches occurred.
"The system experienced nine day period of downtime during the first year due to technical problems at the hospital server that hosted the application. No changes were made to the interventions during the trial period apart from fixing minor bugs."
19-ii) Include qualitative feedback from participants or observations from staff/researchers
No qualitative data was used in this paper.
Results from focus groups/interviews with clinicians will be conducted after completion of the study and reported in separate papers.
Analysis of the content in e-messages between patients and clinicians will also be reported in separate papers.

DISCUSSION

20) CONSORT: Trial limitations, addressing sources of potential bias, imprecision, multiplicity of analyses
20-i) Typical limitations in ehealth trials
"In the recruitment process, most of the potential participants were approached. However, only a third of those approached were included. Lack of access to Internet, the most frequent reason for not meeting the inclusion criteria, was reported by 19% (98/522) of those approached. Among those who were eligible, a frequent reason given for declining participation was that patients judged their computer and Internet skills as poor. One way to increase the participation rates might be to offer a demonstration of the interventions at the time of inclusion."
"A smaller sample size than initially calculated and the attrition rate during the study reduced statistical power for our analyses. Because we had to stop inclusion of participants before the a priori calculated sample was obtained, block randomization lead to different sample sizes in the three groups, with least participants in the IPPC group. The project was also subject to high attrition during the study, which is not uncommon in studies of eHealth interventions (Eysenbach 2005). In addition, the IPPC group had the lowest number of completers of questionnaires at six months. Our analysis should thus be interpreted with caution, and additional research is needed to confirm our results."
"Another limitation relates to low use of the interventions. The analyses of the intervention groups compared to the usual care group therefore compare the effects of a little used intervention. However, as the effects were detected through intention to treat analysis, the effect might be connected to the option and possibility to use the system, not necessarily to actual use. Our post hoc analyses of usage and its relation to outcomes were based on a smaller sample, comparing users and non-users, and must be viewed as an exploratory analysis only."
"Interventions such as WebChoice, that offer components where people interact with each other (forum and blog), need a critical mass in order to be fully utilized. A study period of one year, an inclusion period of nearly two and a half years and the three armed study design resulted in few participants receiving the WebChoice intervention simultaneously. This could be an explanation for low use of the discussion forum in this study."
21) CONSORT: Generalisability (external validity, applicability) of the trial findings
21-i) Generalizability to other populations
"Patients included in the study were younger (median age 52) than those who were excluded (median age 67) or who declined to participate (median age 59). In addition, the participants had higher education levels than the average level of education in Norway, suggesting that they were not representative of all age and educational groups among breast cancer patients. This, together with the small sample size, rate of declining participation and the attrition from the study likely lowers the generalizability of the findings. On the other hand, the patients were recruited from three different hospitals across the country, which increased generalizability of findings across practice settings."

21-ii) Discuss if there were elements in the RCT that would be different in a routine application setting
"Our study illustrates the feasibility of offering parts of Web-based interventions in regular care, as the IPPC component was answered by nurses/physicians at the hospital where the patients were treated., providing the patients with easy access to the expertise, without a face-to-face appointment."
"The integration of Web-based support into clinical practice will require some changes, and changing routines in care is challenging [51-53]. There are reports of skepticism among care providers about use of IPPCs in routine care [54,55]. Some health care providers have expressed concerns that the use of e-messages might disrupt existing workflows and increase workloads [54]. Patients, however, expect to be able to communicate with their health care providers through e-messages [27,54,56], and integration of the IPPC (as a stand-alone service or as part of multi-component support systems) does not require a huge change in health care routines. In this study, only a few nurses and physicians were trained to answer the IPPC. The number of e-messages in the study was moderate and most were answered by nurses, during their regular working hours. As such, the IPPC did not interfere with the workflow of the entire staff, and the number of e-messages was reported as manageable. The nurses answering the IPPC performed their new task during regular working hours, without any incentives. This indicates that it is feasible to implement IPPCs in regular care, and that the service can be managed and answered by nurses. If clinicians recognize Web-based support as effective and easy-to-access resources for their patients' self-management support and outcomes, they might be more receptive to these types of interventions or added service options [57]."
"In the current study, the e-messages were primarily answered by nurses and passed on to physicians only if needed, which indicates that the IPPC can successfully be managed by nurses as the front line."
22) CONSORT: Interpretation consistent with results, balancing benefits and harms, and considering other relevant evidence
22-i) Restate study questions and summarize the answers suggested by the data, starting with primary outcomes and process outcomes (use)

"The current effectiveness study demonstrates that access to the multi component Web-based support system WebChoice for 6 months, among women with breast cancer within the first year after diagnosis, reduced symptom distress and levels of anxiety and depression scores. A tendency towards increased self-efficacy could also be detected for the WebChoice group. This is promising given three diverse practice settings in regular care. Also noteworthy is the finding that access to an IPPC alone reduced depression scores. These results support the hypothesis of the WebChoice group to have better outcomes than the IPPC group in symptom distress and anxiety, compared to the usual care group. The IPPC group had a similar effect on reduction of depression as WebChoice, however, and the WebChoice group did not have better self-efficacy than the IPPC group compared to usual care."

22-ii) Highlight unanswered new questions, suggest future research

"An aspect that remains to be tested is whether Web-based support systems are more effective when health care personnel with treatment responsibilities for the patients answer messages within the system, rather than health care personnel without this knowledge (patients can send e-messages anonymously). To obtain a deeper understanding of experience with the use of IPPC in routine care, we are currently interviewing nurses and physicians who have answered e-messages in this study. This experience is important to guide the implementation processes in the future. In addition, as most studies report on services between patients and physicians, more research is needed to test similar services managed by nurses. Finally, the positive effects on patients' outcomes, despite moderate user frequencies and almost no differences detected between users and non-users, calls for further research examining how the psychological effect of simply having access to information and support might impact outcomes. "

Other information

23) CONSORT: Registration number and name of trial registry

Clinical trial.gov:NCT00971009

24) CONSORT: Where the full trial protocol can be accessed, if available

All documentation is held with study personnel.

25) CONSORT: Sources of funding and other support (such as supply of drugs), role of funders

"The study was funded by the South-East Regional Health Authority of Norway (grant number: 2009051). WebChoice was provided by the Centre for Shared Decision Making and Collaborative Care Research at Oslo University Hospital, Norway. The center delivered the intervention and managed the trial."

X26-i) Comment on ethics committee approval

"The study was approved by the Regional Committee for Medical and Health Research Ethics and the Data Security Inspectorate in Norway.

x26-ii) Outline informed consent procedures

"Eligible patients scheduled for surgery or coming for check-ups after surgery or treatment were identified by the study nurses at the hospitals and provided with information about the study. Upon patients' arrival at the clinic, the study nurses met the patients, provided brief information about the study and asked if they were interested in participating. If the patients agreed, the nurse informed them about the study's purpose and procedures, and asked for written informed consent."

X26-iii) Safety and security procedures

"All data were submitted to a secure server using an encrypted connection. Patients and health care personnel were authenticated using a public key solution that is currently used by Norwegian banks as a security platform. This means the users' log-on procedure is the same whether they log on to their online bank or to IPPC/WebChoice. Thus, patients did not need to learn a new procedure."

"Care providers ….. who answered questions from patients consisted of a dedicated group of expert nurses and physicians in breast cancer care, and social workers at the hospital where the patients were treated. They were thoroughly trained in administering of the IPPC, technically as well as in codes of conduct for online communication with patients. There was a clear schedule for who was responsible for answering patients' messages. The nurses were frontline, and received all messages first. If necessary, they could forward the message to other care providers. If considered important, information from e-messages could be copied into the medical record and made available for other health care providers. When new questions arrived in the system, the recipient was notified through the hospital's e-mail system or by SMS."

"The forum and blog were monitored by nurses at our research center. The nurses did not participate in the forum or blog, but answered two postings in the forum that were not answered by the other participants."

X27-i) State the relation of the study team towards the system being evaluated

"The last author (CR) is the developer of WebChoice, but has no financial or ownership rights to the application. EB, MC, ME and CR are affiliated to the developing Centre, but have no financial or nonfinancial interest to declare in relation to the study."
